# Water-mediated cation intercalation of open-framework indium hexacyanoferrate with high voltage and fast kinetics

**DOI:** 10.1038/ncomms11982

**Published:** 2016-06-20

**Authors:** Liang Chen, Hezhu Shao, Xufeng Zhou, Guoqiang Liu, Jun Jiang, Zhaoping Liu

**Affiliations:** 1 Ningbo Institute of Materials Technology and Engineering, Chinese Academy of Sciences, Ningbo 315201, China

## Abstract

Rechargeable aqueous metal-ion batteries made from non-flammable and low-cost materials offer promising opportunities in large-scale utility grid applications, yet low voltage and energy output, as well as limited cycle life remain critical drawbacks in their electrochemical operation. Here we develop a series of high-voltage aqueous metal-ion batteries based on ‘M^+^/N^+^-dual shuttles' to overcome these drawbacks. They utilize open-framework indium hexacyanoferrates as cathode materials, and TiP_2_O_7_ and NaTi_2_(PO_4_)_3_ as anode materials, respectively. All of them possess strong rate capability as ultra-capacitors. Through multiple characterization techniques combined with *ab initio* calculations, water-mediated cation intercalation of indium hexacyanoferrate is unveiled. Water is supposed to be co-inserted with Li^+^ or Na^+^, which evidently raises the intercalation voltage and reduces diffusion kinetics. As for K^+^, water is not involved in the intercalation because of the channel space limitation.

## Abstract (web summary)

Rechargeable aqueous metal-ion batteries are promising for large-scale applications, but performance and stability issues still remain. Here the authors utilize open-framework indium hexacyanoferrate cathodes to improve performance, showing that water-mediated cation intercalation occurs at the cathode.

## Graphical abstract

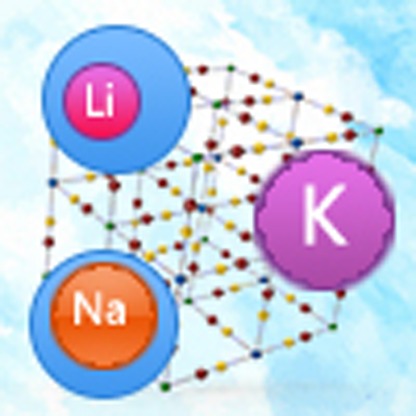

With the increased demand for clean energies such as solar and wind power that are integrated into the utility grid, rechargeable aqueous metal-ion batteries (RAMB) have drawn a great deal of attention because of their better safety, higher-rate capability, lower cost and more eco-friendliness relative to organic counterparts^1^,^2^,^3^. To date, a variety of RAMB on the basis of metal-ion intercalation chemistry have been explored^4^,^5^,^6^,^7^,^8^,^9^,^10^,^11^,^12^,^13^,^14^,^15^,^16^. Particularly, RAMB that use alkali cations (Li^+^, Na^+^ and K^+^) as electrochemical shuttles have garnered great interest^8^,^9^,^10^,^11^,^12^,^13^,^14^. As sodium and potassium are more abundant than lithium, RAMB with Na^+^ and K^+^ shuttles are considered as more competitive power sources for large-scale energy storage. Nevertheless, due to the larger radii of Na^+^ (102 pm) and K^+^ (138 pm) in contrast to Li^+^ (76 pm), a few intercalation compounds, including tunnel-structured oxides, polyanionic phosphates, hexacyanometallates and layer oxides that are suitable for reversible insertion/extraction of Na^+^ or K^+^ in aqueous media have been identified so far^8^,^9^,^10^,^11^,^12^,^13^,^14^,^17^,^18^,^19^,^20^,^21^,^22^. Among them, only NaTi_2_(PO_4_) and NaMn_2_(CN)_6_ can be used as anode materials^11^,^13^. Thus, the known RAMB based on sodium-ion and/or potassium-ion technology are quite limited, and most of them suffer the problem of low-energy density due to the low-voltage output (<1.2 V). Seeking high-voltage RAMB as viable alternatives to commercial aqueous batteries for large-scale energy storage still remains a big challenge.

Recently, we have proposed the fundamental concept of ‘M^+^/N^+^-dual shuttles' to construct RAMB, such as Na_0.44_MnO_2_/TiP_2_O_7_, LiMn_2_O_4_/Na_0.22_MnO_2_, Ni_1_Zn_1_HCF/TiP_2_O_7_ and Ni_1_Zn_1_HCF/NaTi_2_(PO_4_)_3_, which are the so-called aqueous mixed-ion batteries (AMIB)^23^,^24^. Unlike the traditional ‘rocking-chair' lithium-ion battery, such batteries operate based on the migration of dual shuttles between electrode and electrolytes. Their one important characteristic is that the total concentration of M^+^ and N^+^ is fixed during charging/discharging, but the M^+^/N^+^ ratio is changed. Unfortunately, all of them are still limited to low-voltage output (<1.5 V), as well as rapid capacity fading, which greatly hinder their practical application. Interestingly, it is also found that the metal hexacyanoferrate (MeHCF) with open-framework structure allowing for co-insertion/extraction of alkali cations can act as a promising cathode candidate for AMIB. MeHCF with the general formula A_x_M[Fe(CN)_6_]_y_ always belongs to the face-centred cubic structure, in which MC_6_ and FeN_6_ octahedra are bridged through CN ligands. Its unit cell consists of eight CN-bridged transition metal cubes, and provides lots of interstitial sites that can host a wide range of cations, including Rb^+^ and Cs^+^, or even water molecules. Such an open-framework with large interstitial sites enables rapid transport of cations with different ionic radii throughout the lattice. But the fundamental knowledge about how alkali cations are intercalated into MeHCF, especially in aqueous electrolytes, which is of interest scientifically, is still lacking at present. In addition, we and others have found that the electrode potential of MeHCF can be easily tuned by altering the transition metal M, which offers a new routine for viable cathode materials with high voltage and long lifetime to make functional AMIB in the near future^24^,^25^,^26^,^27^,^28^,^29^,^30^.

In this article, we introduce open-framework indium HCF (InHCF) as a cathode material for AMIB. Its unique electrochemical behaviour with various single and dual alkali cations A^+^ are uncovered. The roles of orientation, graphene and guest species on its rate capability are also extensively studied. By combining it with carbon-coated TiP_2_O_7_ and NaTi_2_(PO_4_)_3_ as anode materials, a series of AMIB with high-voltage output ≥1.2 V are demonstrated. All of them possess high power density as an ultra-capacitor, but with higher-energy density. Finally, water-mediated cation intercalation in InHCF is revealed, using multiple characterization techniques coupled with density functional theory (DFT) calculations.

## Results

### Structure of indium hexacyanoferrate

**Figure 1:**
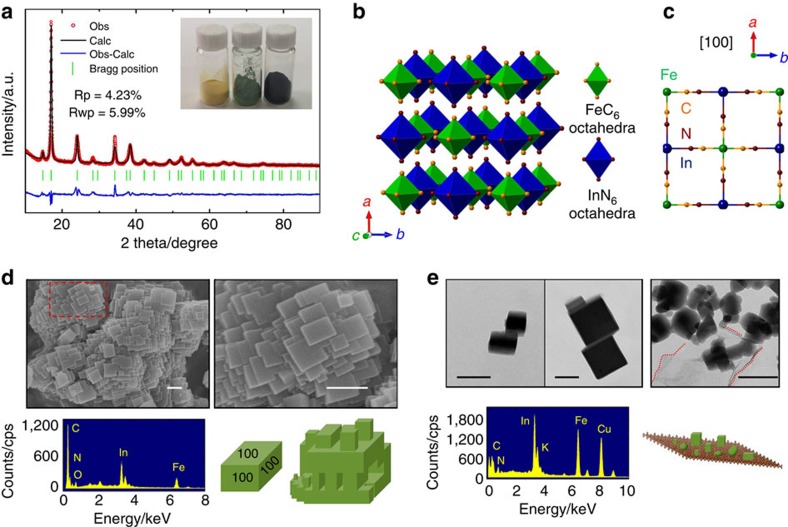
Cubic InHCF. (**a**) Rietveld refinement of X-ray power diffraction pattern for InHCF with cubic structure. Insets are the photos of fresh InHCF, InHCF after vacuum drying and InHCF/Gr. (**b**) A unit cell of cubic InHCF with a formula of InFe(CN)_6_. (**c**) Ball stick pattern of InFe(CN)_6_ viewed down the [100] zone axis. (**d**) SEM images of InHCF nanocube with different magnification (top of panel), energy-dispersive X-ray spectroscopy (EDS) of InHCF nanocubes and a schematic of ‘toy bricks' assembled by InHCF nanocube with six facets of [100] (bottom of panel). Scale bar, 200 nm. (**e**) TEM images of InHCF nanocube and InHCF/Gr (top of panel), EDS of InHCF/Gr (bottom of panel) and a schematic of InHCF with irregular shapes deposited on graphene. Scale bar, 100 nm.

Indium HCF with and without graphene modification (InHCF and InHCF/Gr) were synthesized by a simple precipitation method described in the Methods section below. The fresh InHCF is yellow, and turns into green after vacuum drying. When modified with graphene, it changes into a black solid. X-ray diffraction analyses of above three compounds show that they all exhibit the same face-centred cubic structure as other MeHCFs^9^,^24^,^25^,^31^. Through the Rietveld refinement (Fig. 1a), InHCF is found to adapt the *Fm*-3m space group with a lattice parameter of 10.51 Å, larger than those of NiHCF (10.09 Å), CuHCF (10.1 Å) and FeHCF (10.18 Å)^9^,^25^,^31^. Figure 1b displays its structure with a formula of InFe(CN)_6_, a three-dimensional (3D) framework made up of FeC_6_ octahedra and InN_6_ octahedra linked together by CN ligands. The framework contains open [100] channels with a size of ca. 5.3 Å as shown in Fig. 1c, which allows for a rapid diffusion of a wide variety of guest cations. The ∠Fe−C−N and ∠In−N−C bond angles are both 180°, while the bond distances of Fe−C, In−N and C−N are 1.97, 2.13 and 1.15 Å, respectively. As well, carbon-coordinated Fe (III) in a strong crystal field favours the low-spin configuration (*t*_2g_^5^, *S*=1/2), while nitrogen-coordinated In (III) has zero unpaired electrons at no spin state. With the insertion of alkali cations A^+^, Fe (III) is supposed to be reduced to Fe (II) with zero unpaired electrons (*t*_2g_^6^, *S*=0), while In (III) maintains the same valence. In our DFT calculations, the lattice parameter of InHCF is optimized to be 10.61 Å in excellent agreement with the experimental value (10.51 Å). Other calculated structural parameters including bond distances, bond angles and fractional coordinates summarized in Supplementary Tables 1 and 2 are in accord with the experimental values, suggesting that GGA+U calculations offer adequate accuracy in the description of InHCF.

The morphologies of InHCF and InHCF/Gr are further characterized by scanning electron microscopy (SEM). InHCF looks like ‘toy bricks' assembled by well-crystallized nanocubes with sizes between 50 and 200 nm. Each nanocube with six [100] orientations shows a single-crystalline state (Fig. 1d). Elemental analyses show that In, Fe and N elements with atomic ratios of 15:21:64 are found. From their transmission electron microscopy (TEM) images, cube-like nanoparticles with sizes ranging from 50 to 200 nm are also observed (Fig. 1e), which are consistent with SEM characterizations. Periodic diffraction spots taken from a single particle correspond to a single crystal with [100] orientations (Supplementary Fig. 1). However, irregular nanoparticles randomly deposited on graphene are observed for InHCF/Gr (Fig. 1e; Supplementary Fig. 1). The average particle size is ca. 50 nm, smaller than that of InHCF. It is anticipated that two-dimensional graphene can adsorb InHCF nanocrystals during the nucleation stage of precipitation reaction, which blocks their further growth up. Elemental analysis from TEM also shows that In, Fe and N elements coexist in InHCF/Gr.

### Electrochemical behaviour of InHCF

**Figure 2:**
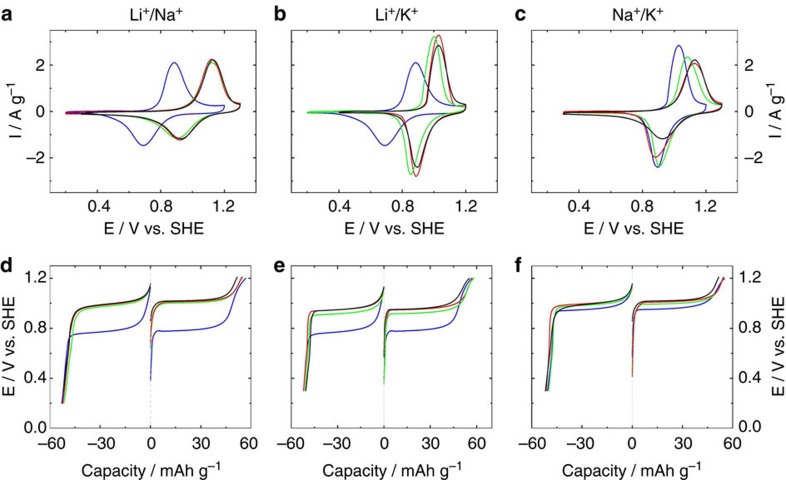
Electrochemical properties of InHCF/Gr. (**a**–**f**) Cyclic voltammograms (**a**–**c**) at a scan rate of 2 mV s^−1^ and galvanostatic profiles (**d**–**f**) measured at a rate of 1C for InHCF/Gr in various electrolytes (1C=60 mA g^−1^). All electrode potentials are versus standard hydrogen electrode (SHE). Blue line is corresponding to 0.5 M Li_2_SO_4_ in **a**,**b**,**d** and **e**) or 0.5 M K_2_SO_4_ in **c** and **f** Green line is corresponding to 0.25 M Li_2_SO_4_/0.25 M Na_2_SO_4_ in **a** and **d** or 0.25 M Li_2_SO_4_/0.25 M K_2_SO_4_ in **b** and **e** or 0.25 M Na_2_SO_4_/0.25 M K_2_SO_4_ in **c** and **f**. Red line is corresponding to 0.1 M Li_2_SO_4_/0.4 M Na_2_SO_4_ in **a** and **d** or 0.1 M Li_2_SO_4_ /0.4 M K_2_SO_4_ in **b** and **e** or 0.4 M Na_2_SO_4_/0.1 M K_2_SO_4_ in **c** and **f**. Black line is corresponding to 0.5 M Na_2_SO_4_ in **a**,**c**,**d** and **f** or 0.5 M K_2_SO_4_ in **b** and **e**.

The electrochemical behaviour of electrodeposited MeHCF thin films with a variety of anions have been investigated by Neff's and other groups since 1980s (refs 32, 33, 34). However, such thin films have mass loading as low as several μg cm^−2^, which is too far from their practical battery application. Herein, our bulk InHCF electrodes have mass loadings of ca. 10 mg cm^−2^, 100 times over previous electrodeposited films. As shown in Fig. 2a–c, InHCF/Gr exhibits one pair of well-defined and symmetric redox peaks with either alkali cations A^+^ (Li^+^, Na^+^ or K^+^). Such characteristic peaks are associated with the reversible conversion between Fe(II) and Fe(III) accompanied by the storage/release of A^+^, which are demonstrated in the Discussion section below. The formal potential *E*_f_ follows the order: Li^+^ (0.79 V)<K^+^ (0.97 V)<Na^+^ (1.03 V). This trend is different from CuHCF, NiHCF and Ni_x_Zn_y_HCF, where the intercalation of A^+^ with larger ionic radius happens at higher voltage (Li^+^<Na^+^<K^+^)^12^,^24^. Δ*E*_p_ (the difference between anode and cathode peak potentials, *E*_pa_−*E*_pc_) is an important factor for energy loss in a battery, and small Δ*E*_p_ means low polarization overpotential and fast reaction kinetics. At a scan rate of 2 mV s^−1^, Δ*E*_p_ of InHCF/Gr with A^+^ are within 200 mV, indicating a fast reaction between Fe(II) and Fe(III). And Δ*E*_p_ follows another order as *E*_f_: 130 mV (K^+^)<180 mV (Li^+^)<200 mV (Na^+^). Furthermore, a specific capacity of 52±1 mAh g^−1^ (1C rate) is measured for InHCF/Gr with either A^+^, showing that it has the same storage ability with alkali cations, regardless of their different ionic radii (Fig. 2d–f; Supplementary Fig. 2 and Supplementary Note 1). Its rate capability with A^+^ follows the opposite order as Δ*E*_p_ :K^+^>Li^+^>Na^+^. The high-rate capacity retention of InHCF/Gr is greatest when it is cycled with K^+^ for its 93% capacity retention at 10C and 79% at 40C (Supplementary Fig. 3). Intercalation of Li^+^ and Na^+^ into InHCF/Gr occurs at moderate high rates, as it retains 64 and 44% of its discharge capacity at 40C during their respective reactions. In addition, the rate behaviours of InHCF without graphene modification are also tested for comparison. It is found to be less impressive, with 59% (Li^+^), 37% (Na^+^) and 73% (K^+^) of the discharge capacities retained at 40C in those cases (Supplementary Fig. 3).

The electrochemical behaviours of InHCF/Gr in mixed-ion electrolytes are also revealed. In the case of Li_2_SO_4_/Na_2_SO_4_ electrolytes, one pair of redox peaks still appears. But their potentials are close to the ones in Na_2_SO_4_. Meanwhile, the charge/discharge voltage plateaus in mixed-electrolytes are also close to the ones in Na_2_SO_4_, which are about 200 mV higher than the ones in Li_2_SO_4_. The discharging capacities in mixed-electrolytes are ∼52 mAh g^−1^ (1C rate), identical to the one in Li_2_SO_4_ or Na_2_SO_4_. The *E*_ocp_ and *E*_1/2_ increase with an increase of Na^+^ concentration in electrolytes (Supplementary Fig. 4), implying that the existence of Na^+^ gives rise to high working voltage, which is desirable for high-energy density. Moreover, the capacity retentions of InHCF/Gr at 40C in 0.25 M Li_2_SO_4_+0.25 M Na_2_SO_4_ and 0.1 M Li_2_SO_4_+0.4 M Na_2_SO_4_ are 49 and 51%, between those in 0.5 M Li_2_SO_4_ and 0.5 M Na_2_SO_4_ (Supplementary Figs 5–6). Obviously, the introducing of Li^+^ into Na_2_SO_4_ electrolytes is beneficial to the rate capability of InHCF/Gr.

With regard to Li_2_SO_4_/K_2_SO_4_ electrolytes, similar behaviours are observed. Figure 2b displays that the *E*_pa_ and *E*_pc_ in Li_2_SO_4_/K_2_SO_4_ electrolytes are higher than those in Li_2_SO_4_, while the Δ*E*_p_ becomes smaller. Moreover, the charge/discharge voltage plateaus in Li_2_SO_4_/K_2_SO_4_ are ∼150 mV higher than those in Li_2_SO_4_. Both *E*_1/2_ and *E*_ocp_ are electrolytes dependent, which go up with an increase of K^+^ concentration in electrolytes (Supplementary Fig. 4). In addition, the capacity retention of InHCF/Gr at 40C in Li_2_SO_4_/K_2_SO_4_ electrolytes is up to 85%, higher than those in Li_2_SO_4_ and K_2_SO_4_ (Supplementary Fig. 5–6). It is illustrated that the addition of K^+^ into Li_2_SO_4_ electrolytes can enhance both the working voltage and rate capability of InHCF/Gr.

In Na_2_SO_4_/K_2_SO_4_ electrolytes, these behaviours are not as notable as those in Li_2_SO_4_/Na_2_SO_4_ and Li_2_SO_4_/K_2_SO_4_ electrolytes. When InHCF/Gr is cycled in 0.4 M Na_2_SO_4_/0.1 M K_2_SO_4_ (1C rate), the discharge voltage plateau is almost identical to that in 0.5 M Na_2_SO_4_, which are 50 mV higher than that in 0.5 M K_2_SO_4_. At the same time, the polarization (voltage difference between charging and discharging profiles) is only 16 mV at 1C, smaller than that in Na_2_SO_4_ (32 mV). When it is cycled at 40C, the capacity retention remains 64% much higher than 44% in Na_2_SO_4_ (Supplementary Figs 5–6). These results demonstrate that the existence of K^+^ in Na_2_SO_4_ electrolytes can alleviate the polarization without the loss of the working voltage.

### Aqueous mixed-ion batteries

**Figure 3:**
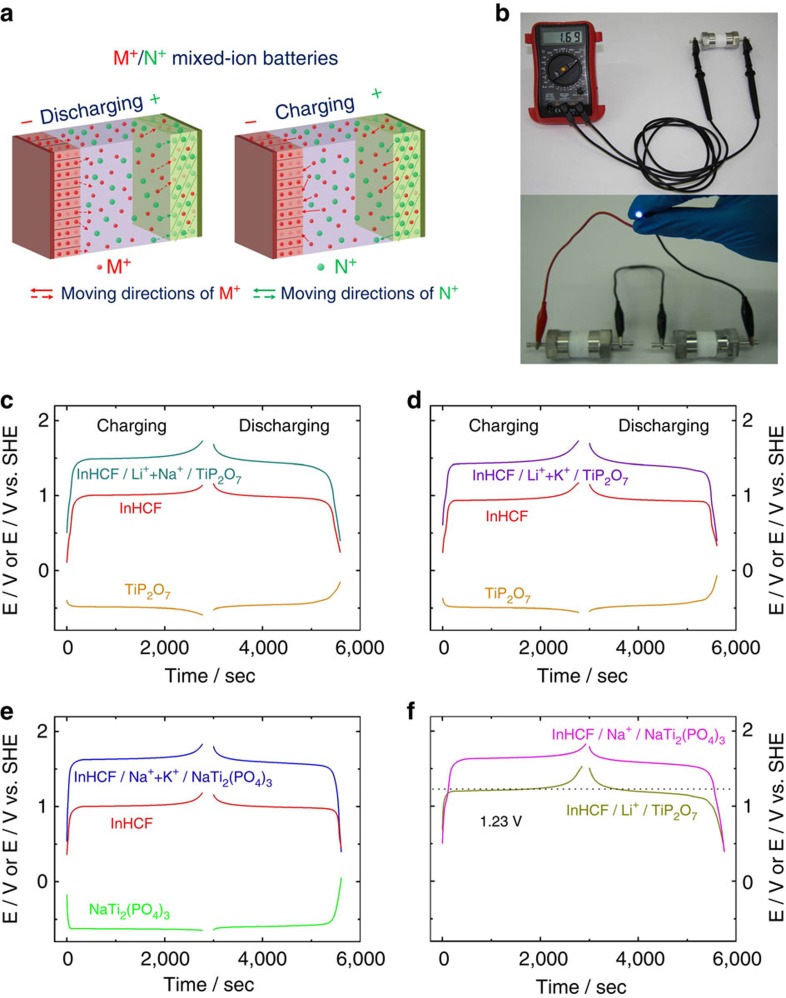
Aqueous mixed-ion batteries. (**a**) A schematic of M^+^/N^+^ aqueous mixed-ion battery. (**b**) Photos of InHCF/Na^+^+K^+^/NaTi_2_(PO_4_)_3_ (B-III) battery in a Swagelok-type cell. One is connected with a voltage metre, and two in series are lighting up a 3-V blue LED. (**c**–**e**) Galvanostatic profiles of InHCF/Li^+^+Na^+^/TiP_2_O_7_ (B-I), InHCF/Li^+^+K^+^/TiP_2_O_7_ (B-II) and InHCF/Na^+^+K^+^/NaTi_2_(PO_4_)_3_ (B-III) batteries along with the voltage profiles of their individual anode and cathode electrodes versus SHE at a rate of 1.5C. (**f**) Galvanostatic profiles of InHCF/Li^+^/TiP_2_O_7_ (B-IV) and InHCF/Na^+^/NaTi_2_(PO_4_)_3_ (B-V) batteries measured at a rate of 1.5C. 1C=60 mA g^−1^.

On the basis of the unique intercalation chemistry of InHCF/Gr, we assembled three practical prototypes of batteries operating in mixed-ion electrolytes (B-I, B-II and B-III). Figure 3a illustrates the principle of such batteries: the release and storage of M^+^ with small ionic radius occur at anode, and the co-insertion/extraction reactions of M^+^ and N^+^ (large ionic radius) take place at cathode. During charging/discharging, the total concentration of M^+^ and N^+^ is fixed to ensure the charge neutrality of the electrolytes, but the M^+^/N^+^ ratio is changed. Our previous studies show that the cubic TiP_2_O_7_ and rhombohedral NaTi_2_(PO_4_)_3_ can be used as the anodes for AMIB, as a result of their specific ion-selectivity properties and reasonable working voltages^23^,^24^. Herein, carbon-coating TiP_2_O_7_ and NaTi_2_(PO_4_)_3_ synthesized by solid-state reactions are selected as the anodes for B-I (InHCF/Li^+^+Na^+^/TiP_2_O_7_), B-II (InHCF/Li^+^+K^+^/TiP_2_O_7_) and B-III (InHCF/Na^+^+K^+^/NaTi_2_(PO_4_)_3_). Through X-ray diffraction and Rietveld refinements, a pure cubic phase for TiP_2_O_7_ and a pure rhombohedral phase for NaTi_2_(PO_4_)_3_ are obtained (Supplementary Figs 7–8). High-resolution TEM (HR-TEM) analyses show that uniform carbon layers with a thickness of ca. 10 nm are coated on them, ensuring their good electrochemical performances (Supplementary Figs 7–8). The galvanostatic profiles of B-I, B-II and B-III along with the voltage profiles of their individual anode and cathode electrodes versus standard hydrogen electrodes (SHE) are displayed in Fig. 3c–e. An average working voltage (*E*_av_) is found to be 1.45 V for B-I, 1.4 V for B-II and 1.6 V for B-III, which is beyond a theoretical water electrolysis voltage of 1.23 V. The galvanostatic profiles of InHCF/Li^+^/TiP_2_O_7_ (B-IV) and InHCF/Na^+^/NaTi_2_(PO_4_)_3_ (B-V) batteries are added for comparison (Fig. 3f). Their *E*_av_s are only 1.2 and 1.55 V, lower than those of their corresponding AMIB. In Supplementary Table 3, it shows that B-I, B-II and B-III compare favourably with our previous AMIB and many other RAMB, in terms of *E*_av_ and energy density. In particular, *E*_av_ of B-III, as well as B-V is up to 1.6 V, which has been the highest record among RAMB based on sodium-ion technology until now.

**Figure 4:**
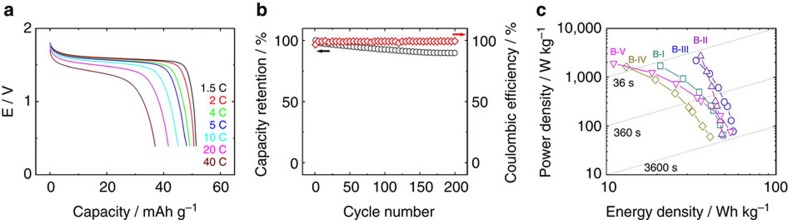
Rate capability and cycle life of mixed-ion batteries. (**a**) Rate capability of B-III battery. (**b**) Long-term cycling performance for B-III battery at a rate of 1.5C. (**c**) A Ragone plot of B-I, B-II, B-III, B-IV and B-V.

In addition to high *E*_av_, B-III also possesses strong high-rate capability. At current rates as high as 40C, the discharge capacity remains >73% of that at 1.5C (Fig. 4a). As well, its cycle life is good. During galvanostatic cycling between 0.4 and 1.83 V at 1.5C, ∼90% of the initial discharge capacity are retained after 200 cycles (Fig. 4b). The coulombic efficiency is ∼100% during cycling except the first cycle, suggesting that many side reactions (for example, water electrolysis reactions) are eliminated during cycling despite the high-voltage charge limit (1.83 V) for B-III (Supplementary Fig. 9; Supplementary Table 4; Supplementary Notes 2 and 3). A summary of the discharged energy and power derived from B-III along with B-I and B-II are displayed in Fig. 4c. A specific energy of 49, 48 and 56 Wh kg^−1^ based on the total mass of active electrode materials are obtained for B-I, B-II and B-III, respectively. Typically, the electrode materials are ∼ 60% of the total weight of the practical cells with large size^4^,^7^. Thus, a practical specific energy of B-III close to 34 Wh kg^−1^ can be expected, which can compete with current Pb-acid battery with ca. 35 Wh kg^−1^ for energy storage. They also demonstrate high power density comparable to that of an ultra-capacitor (>1,000 W kg^−1^), but with much higher-energy density (>20 Wh kg^−1^). For instance, B-II can deliver a specific power of 2,700 W kg^−1^ at a specific energy of 36 Wh kg^−1^. Their capacity retentions at various rates follow the same order as InHCF/Gr: B-II (Li^+^/K^+^)>B-III (Na^+^/K^+^)>B-I (Li^+^/Na^+^), implying that B-II is the most promising system to meet high power energy storage among them. The Ragone plots of B-IV and B-V are also displayed in Fig. 4c. As seen, their performances are much less impressive, when compared with their corresponding AMIB.

### Roles of orientation, graphene and mixed-ion electrolytes

Both the dimensionality and activation energy of cation migration within the crystal structures of electrode materials are critical for their rate capabilities. The high-rate capability of InHCF arises from its special structure. InHCF is composed of nanocubes with six [100] facets, which can behave as 3D diffusion pathways for A^+^ migration. Meantime, A^+^ immigration through [100] facets is supposed to have low activation energy, due to the large open space of [100] channel. Compared with InHCF, the higher-rate capability for InHCF/Gr is ascribed to another two factors. One is that graphene in InHCF/Gr serves as a 3D electronic network to diminish the resistance between InHCF nanoparticles, which facilitates the electron transfer (Supplementary Note 4). The other is size effect of InHCF/Gr whose average size is smaller than that of InHCF, giving shorter diffusion path for A^+^ migration (Supplementary Fig. 10).

Owning to the unique electrochemical properties of InHCF/Gr cathode in mixed-ion electrolytes as previously described, the utilization of mixed-ion electrolytes have two merits: raising the reaction potential (*E*) and speeding up reaction kinetics. For a battery cathode, raising *E* can lead to high-voltage output that is desirable for high-energy density. When an intercalation reaction occurs at H with ion-selectivity towards A^+^ as shown in equation (1), *E* can be calculated using the Nernst equation (2):


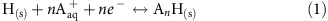



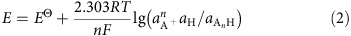


where 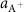, *a*_H_ and 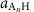 refer to the activities of A^+^, H and A^+^-intercalated H (A_*n*_H). From equation (2), *E* is found to be strongly dependent on A^+^ activity. For a battery anode, reducing *E* can expand the working voltage that is preferable for high-energy density. Using mixed-ion electrolytes that can decrease A^+^ activity 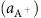 is a good tool for reducing *E*, which has been demonstrated on TiP_2_O_7_ and NaTi_2_(PO_4_)_3_ materials in our previous works. Therefore, utilizing mixed-ion electrolytes instead of one single electrolyte for InHCF/TiP_2_O_7_ and InHCF/NaTi_2_(PO_4_)_3_ batteries have two merits: one is to raise the voltage output and the other is to enhance the rate capability, which have been demonstrated by the electrochemical data (Supplementary Note 5).

### Water-mediated cation intercalation in InHCF

**Figure 5:**
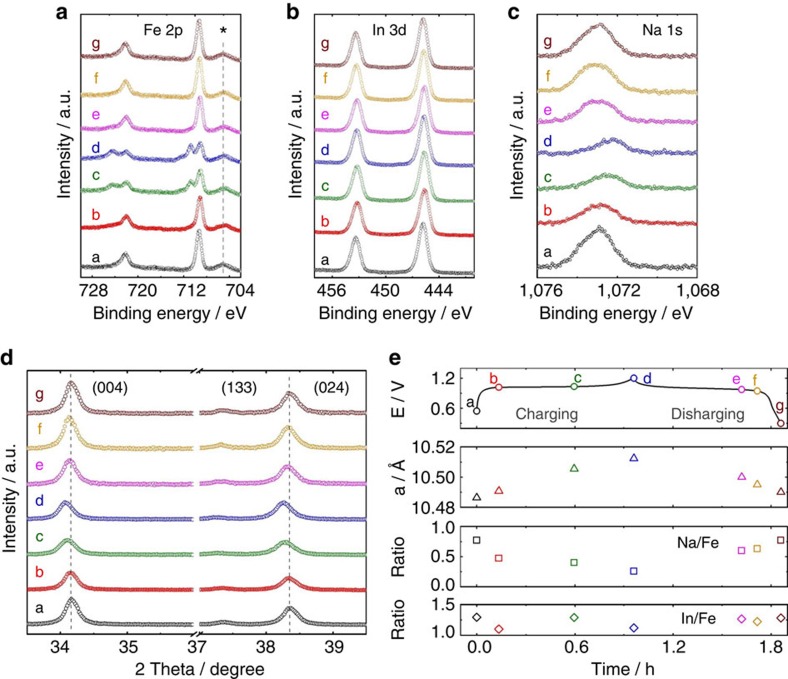
Sodium-ion intercalation mechanism in InHCF. (**a**–**c**) *Ex situ* XPS spectra recorded from Fe 2*p*, In 3*d* and Na 1*s* core level of InHCF/Gr upon electrochemical cycling (a–g states as shown in **e** are selected). A binding energy of 688.2 eV for the F 1*s* (from polyvinylidene fluoride (PVDF)) was used as reference. The peaks labelled by * symbol (705.0±0.1 eV) are related to In 3*p*_1/2_. (**d**) *Ex situ* X-ray diffraction patterns of InHCF/Gr with cycling. (**e**) Changes of lattice parameter (*a*), surface atomic ratio (Na/Fe and In/Fe) in InHCF/Gr during electrochemical cycling. a, 0.55 V, b, 1.02 V, c, 1.035 V, d, 1.2 V, e, 0.975 V, f, 0.945 V and g, 0.3 V seven states are selected for comparison.

Due to the highest intercalation voltage of Na^+^ among A^+^, the Na^+^-intercalation reaction is selected as a probe to understand the intercalation mechanisms of alkali cations in InHCF. First, we used *ex situ* X-ray photoelectron spectroscopy (XPS) to analyse the variations of oxidation states of Fe, In and Na elements in InHCF/Gr upon electrochemical cycling. Fe 2*p* core level of the material at a−g states are displayed in Fig. 5a. The peaks at 709.2 and 722.1 eV for InHCF/Gr can be attributed to the 2*p*_3/2_ and 2*p*_1/2_ of Fe (II). During charging (from a to d), the intensities of these peaks decrease, and two new peaks at 710.8 and 724.4 eV are found for InHCF/Gr with c and d states. The appearances of the new peaks are related to Fe(III). During discharging, these new peaks for InHCF/Gr with e, f and g states are diminished, and the Fe(II) 2*p* peak intensities increase as InHCF/Gr is discharged from d to g. So it is concluded that the reversible redox reactions between Fe(II) and Fe(III) take place during the entire charging/discharging process. *Ex situ* XPS of In 3*p*_1/2_ and 3*d* were also collected (Fig. 5a,b). The peaks centred at 705.0, 445.7 and 453.3 eV, respectively, can be ascribed to the 3*p*_1/2_, 3*d*_5/2_ and 3d_3/2_ of In(III). Unlike Fe 2*p*, all spectra remain almost the same, indicating that In maintain the same valence upon cycling. Similar results were obtained for N 1*s* and F 1*s*, implying that N and F elements like In element do not undergo redox reactions upon cycling (Supplementary Fig. 11)^35^. Meanwhile, the Na 1*s* peak density as shown in Fig. 5c decreases from a to d, and increases from d to g. It is inferred that Na^+^ extraction/insertion reactions happen during their respective charging/discharging process. The atomic ratios of different elements upon cycling determined by XPS are also summarized in Fig. 5e and Supplementary Table 5. As shown, the Na/Fe ratio decreases from 0.77 to 0.26 during charging, and then goes back to original 0.77 after discharging. However, during the electrochemical cycle, the In/Fe and N/Fe atomic ratios remain within 1.23±0.08 and 6.37±0.38, respectively. Compared with In, Fe and N, the obvious composition changes for Na in InHCF/Gr are linked to the Na^+^-insertion/extraction reaction during the cycle. All the above XPS results illustrate that Fe is the redox centre of InHCF that involves the reversible conversion between Fe(II) and Fe(III) accompanying the extraction/insertion of Na^+^.

X-ray diffraction was also employed to monitor the structural characteristics of InHCF/Gr with cycling. As shown in Fig. 5d, InHCF/Gr maintains its cubic structure upon cycling, suggesting a quasi-solid-solution reaction. Interestingly, the X-ray diffraction reflections (for example, (004), (133) and (024)) are found to shift to smaller angles during charging (Na^+^-extraction) and move to higher angles during discharging (Na^+^-insertion). For InHCF/Gr, the lattice contraction induced by Na^+^-insertion is opposite to the lattice expansion of other intercalation compounds, such as graphite, LiFePO_4_, TiP_2_O_7_ and Na_0.44_[Mn_1-x_Ti_x_]O_2_ caused by cation intercalation. But InHCF/Gr undergoes a very small lattice contraction (from 10.51 to 10.49 Å, only ∼0.2%) after Na^+^-insertion, which is close to zero-strain characteristic. Such a small lattice contraction is mainly linked to the slightly decrease in the Fe−C bond distance during reduction, which is confirmed by our DFT calculations (from 1.91 to 1.89 Å). And it is also associated with the measured small polarization and good cycle life for InHCF/Gr.

**Figure 6:**
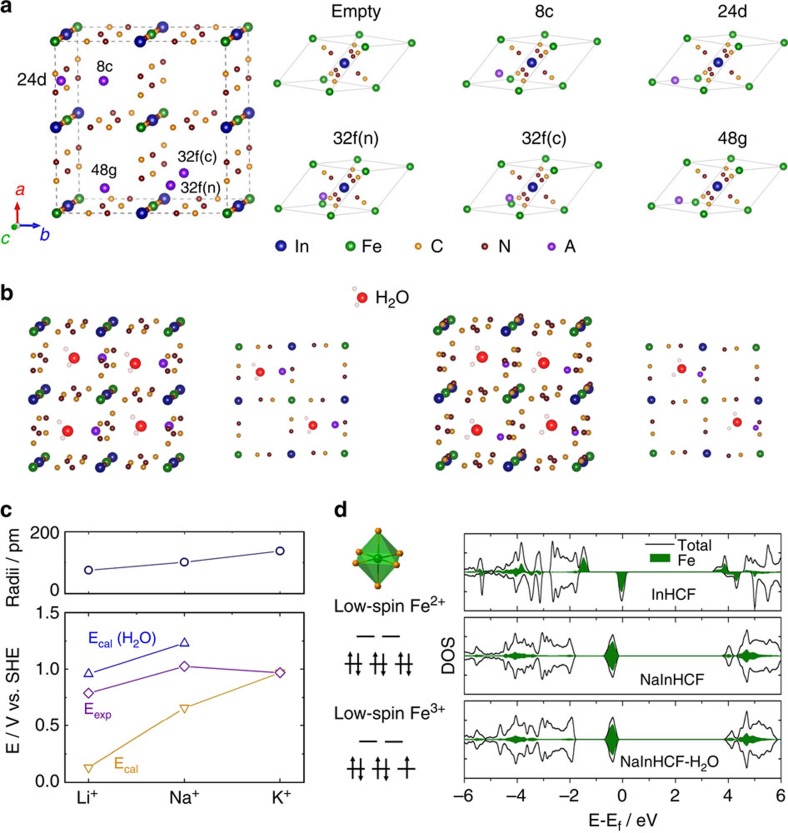
DFT calculations. (**a**) Crystal structure of InHCF with five possible interstitial sites and primitive cells of InHCF with A^+^ occupying the empty, 8c (body centre), 24d (face centre), 32f(n) (displaced from 8c sites toward N-coordinated corner), 32f(c) (displaced from 8c sites toward C-coordinated corner) and 48g (displaced between 8c and 24d) sites. (**b**) Crystal structures of InHCF with intercalation of [Na-OH_2_]^+^ (left of panel) and [Li-OH_2_]^+^ (right of panel). (**c**) Comparison of ionic radii and intercalation voltage for Li^+^, Na^+^ and K^+^. (**d**) Schematics of the electronic states of Fe at different oxidation states and spin states, and electronic density of states projected on Fe ions in InHCF, NaInHCF and NaInHCF-H_2_O.

In the cubic structure of InFe(CN)_6_ as shown in Fig. 6a, A^+^ can occupy five possible interstitial sites, which are denoted with Wyckoff notations as 8c, 24d, 32f(n), 32f(c) and 48g. Among them, 24d site provides the smallest free space, while 8c site gives the largest free space. To avoid significant expansion of the lattice framework, the size of inserted ions to occupy the 24d site and 8c site should be limited to 140 and 260 pm, respectively. To assess the relative stability of the interstitial sites that occupied by different A^+^, the binding energy (*E*_b_) are calculated using DFT (Supplementary Methods)^31^,^36^,^37^,^38^. In terms of Li^+^ and Na^+^, 24d and/or 48g sites give the lowest *E*_b_, while 8c site gives the highest *E*_b_ in Table 1. This trend changes for large cation K^+^. It prefers to occupy 8c site or 48g site in comparison with 24d site. The reason is that small cations (Li^+^ and Na^+^) are more stable at 24d and/or 48g for shorter cation–anion-bonding distances, while large cation K^+^ is less favourable to occupy 24d site because of the space limitation. Another observation is that the 32f(n) site is always more energetically stable than the 32f(c) site, and the nearest A−N distance is always smaller than the A−C ones. It can be interpreted as the electronegative nitrogen is more attractive to A^+^ than carbon. The calculated volumes of the AInFe(CN)_6_ unit with A^+^ occupying different sites (primitive cell) are also summarized in Supplementary Fig. 12. It can be seen that almost all A^+^-intercalation tend to shrink the volume except K^+^-intercalation into 24d site. This can be probably because K^+^ in 24d site has strong steric repulsion with its surrounding atoms, resulting in volume expansion. When considering the most stable interstitial site, the volume contractions after the intercalation of Li^+^, Na^+^ and K^+^ are within 3%, 0.8% and 1%, respectively, which are close to zero-strain characteristic. Such a phenomenon is consistent with our above X-ray diffraction analysis.

It is previously mentioned that a variety of intercalants, including alkali cations and H_2_O can be intercalated into InHCF. However, as a result of the large size of H_2_O (3 Å), the stable interstitial sites for H_2_O are quite limited. Among them, 8c site is ideal for H_2_O occupation, owning to its largest free space. Our DFT calculation shows that the *E*_b_ between H_2_O and InHCF is −0.67 eV, suggesting their much weaker interaction than *E*_b_ between A^+^ and InHCF (Supplementary Fig. 13). As small A^+^ occupying the 24d site in the lattice, the empty 8c site is supposed to accommodate H_2_O, in which O atom has coulomb attraction with positively charged A^+^. To confirm the assumption, a water molecule and A^+^ are initially put into 8c site and 24d site, respectively. After geometry optimization, H_2_O almost stay at original 8c site and A^+^ move to another site displaced from 24d site. When the sites of H_2_O and A^+^ are exchanged by each other, it is also found that after geometry optimization, H_2_O moves from 24d site to a site near 8c site and A^+^ moves towards 24d site. The distance between Na and O (or Li and O) is found to be 2.24 Å (or 1.88 Å), indicative of the interaction between Na^+^ (or Li^+^) and H_2_O (Fig. 6b). *E*_b_([Na-OH_2_]^+^) and *E*_b_([Li-OH_2_]^+^) are calculated to be −3.94 and −4.0 eV, respectively, which are 0.61 and 0.87 eV lower than their corresponding *E*_b_ at 24d site. The more remarkable interaction between Li^+^ and H_2_O is due to the higher ionic potential (charge/radius) of Li^+^ relative to Na^+^. All above results suggest that in terms of Li^+^ and Na^+^, H_2_O can be co-inserted into InHCF with them. As for K^+^, the size of [K-OH_2_]^+^ is up to 288 pm that exceeds the size limit of 8c site, which infers that K^+^ is solely inserted into InHCF without water (Supplementary Fig. 14; Supplementary Note 6). The rate capability of intercalation compounds that critically depends on cation diffusion is related to cation size. It is well known that large cation always suffers from slow migration kinetics, leading to low-rate capability. The sizes of cations follow the order: *r*(K^+^)<*r*(Li-OH_2_^+^)<*r*(Na-OH_2_^+^), providing a good explanation for why the rate capability of InHCF in aqueous electrolytes follow the order: K^+^>Li^+^>Na^+^. In mixed-ion electrolytes, co-insertion/extraction of two alkali cations takes place at cathode side. So it is not surprising that the rate capabilities of InHCF/Gr with Li^+^/Na^+^, Li^+^/K^+^ and Na^+^/K^+^ are better than those with Na^+^, Li^+^ and Na^+^, respectively. Through the equation *V*=−*E*_b_*/ne* (*n*=1 in this case), we can calculate the intercalation potential *V*(Li-OH_2_^+^)=4.0 V versus Li/Li^+^, *V*(Na-OH_2_^+^)=3.94 V versus Na/Na^+^ and *V*(K^+^)=3.86 V versus K/K^+^. When normalized to SHE, *V*(Li-OH_2_^+^)=0.96 V, *V*(Na-OH_2_^+^)=1.23 V and *V*(K^+^)=0.97 V are obtained (Fig. 6c). These values agree well with our experimental *E*_f_s (0.79, 1.03 and 0.97 V versus SHE). Clearly, the calculated and experimental intercalation potential (*V*) follow the same order: *V*(Li-OH_2_^+^)<*V*(K^+^)<*V*(Na-OH_2_^+^). This trend is quite a striking result. In typical intercalation compounds for organic batteries, the voltage to intercalate Na^+^ is ∼0.1–0.5 V lower than that for the intercalation of Li^+^, due to the weaker bonding between Na^+^ and host, while the voltage to intercalate K^+^ can be comparable to that of Li^+^ (ref. 37).

The intercalation mechanism is further verified by analysing the electronic structure of InHCF before and after cation insertion. As illustrated in Fig. 6d, Fe^3+^ in InHCF has one unpaired electron at low-spin state, while Fe^2+^ in AInHCF has zero unpaired electrons. Therefore, the magnetic momentum of Fe atom correlates to its oxidation state and spin state. In InHCF, the calculated magnetic momentum is 1.0 μB for low-spin Fe^3+^. When InHCF changes into AInHCF, the magnetic momentum of Fe atom reduce to 0 μB, corresponding well to the reduction from Fe^3+^ (*t*_2g_^5^) to Fe^2+^ (*t*_2g_^6^). Here, the density of states projected on atomic orbitals (density of states projected on atomic orbitals (PDOS)) for InHCF, NaInHCF and NaInHCF-H_2_O are shown as examples. From InHCF to NaInHCF, the major change of the PDOS is observed for Fe atom, while the main characteristic of In atom is almost unaltered (Supplementary Fig. 15). This result coincides with our XPS examination. The PDOS of Fe and In atoms in NaInHCF-H_2_O are almost identical to those in NaInHCF, implying that water has little effect on the chemical environments of Fe and In atoms, resulting in a weak interaction with InHCF. In short, the experimental and theoretical studies on the intercalation mechanism suggest that in terms of the intercalation compounds for RAMB, the role of water that has been ignored before should be put into consideration.

## Discussion

In summary, a series of high-voltage AMIB (B-I, B-II and B-III) based on unique electrochemical properties of InHCF, TiP_2_O_7_ and NaTi_2_(PO_4_)_3_ in mixed-ion electrolytes are validated. They have high power density comparable to that of an ultra-capacitor, but with much higher-energy density. Among them, B-III exhibits a specific energy of 56 Wh kg^−1^ with *E*_av_ of 1.6 V, and B-II deliver a specific power of 2700 W kg^−1^ as an ultra-capacitor. The cation intercalation chemistry in InHCF is also unveiled. Iron not indium atom in InHCF is confirmed as the centre involved in the redox reaction. Such a reaction is accompanied by the intercalation of alkali cations that induces negligible structural deformations (zero-strain characteristic). DFT calculations also show that the intercalation is strongly affected by the ionic radii of inserted species. In terms of Li^+^ or Na^+^ with small size, they are supposed to be co-inserted into InHCF with H_2_O, while H_2_O occupying the site near 8c site in InHCF. K^+^ with large size is thought to be solely inserted into the 8c site without water, as a result of the space limitation in the [100] channel of InHCF. The calculated voltages for the intercalation of A^+^ are in good agreement with our experimental values. Water-mediated cation intercalation also explains why the rate capability of InHCF with A^+^ in aqueous electrolytes follows the order: K^+^>Li^+^>Na^+^. Overall, our studies not only enrich the family of RAMB, but also offer a supplement to the existing intercalation chemistry, broadening our horizons for battery research.

## Methods

### Material syntheses

Graphene was obtained through thermal exfoliation of graphene oxide (Hummer's method) at 800 °C. InHCF/Gr was synthesized by *in situ* deposition method from our previous literature^16^,^24^. Typically, 100 ml of 0.085 M InCl_3_ (pH 3.0, adjusted by aq. HCl) and 100 ml of 0.043 M K_3_Fe(CN)_6_ were simultaneously added into 75 ml of graphene water suspension (1 mg ml^−1^) under vigorous stirring. The dropping rates of InCl_3_ and K_3_Fe(CN)_6_ solutions were precisely controlled by peristaltic pump (0.6 ml min^−1^). After the reaction was completed, the black InHCF/Gr slurry was formed. Finally, the precipitates were washed with de-ionized water several times and then dried at 80 °C overnight. Orange precipitates were obtained for InHCF by using de-ionized water instead of graphene suspension. TiP_2_O_7_ and NaTi_2_(PO_4_)_3_ were prepared by solid-state reaction method. The high purity TiO_2_ (up to 99.9%) and NH_4_H_2_PO_4_ in a 1:2 ratio were added into ethanol to form paste. This paste was ball-milled in a planetary ball mill at 400 r.p.m. for 6 h. After ball milling, the mixture was dried at 80 °C to evaporate ethanol. Then it was successively heated for 5 h at 300 °C and for 12 h at 900 °C under air with intermediate grindings. The obtained white TiP_2_O_7_ solids, referred as as-prepared TiP_2_O_7_, were homogenized in a mortar for further use. For carbon-coated TiP_2_O_7_, 2.5 g of as-prepared TiP_2_O_7_, 2 g of glucose and appropriate amount of ethanol were mixed and then ball-milled for 4 h. The solids were obtained by evaporating the ethanol at 80 °C. After calcined at 800 °C for 3 h in a mixture gas (C_2_H_4_/Ar, 5/95), the solids became black, implying that TiP_2_O_7_ were coated by carbon. Carbon-coated NaTi_2_(PO_4_)_3_ are prepared by the similar method for carbon-coated TiP_2_O_7_ as stated above, except using the different precursor for NaTi_2_(PO_4_)_3_ (Na_2_CO_3_:TiO_2_:NH_4_H_2_PO_4_=1:4:6).

### Characterization

Powder X-ray diffraction patterns were collected using an AXS D8 Advance diffractometer (Cu Kα radiation; receiving slit, 0.2 mm; scintillation counter, 40 mA; 40 kV) from Bruker Inc. The morphology and structure of samples were analysed by a Hitachi S-4800 field emission SEM and an FEI Tecnai G2 F20 TEM at an accelerating voltage of 200 kV. Thermal gravimetric analysis was performed on a Pyris Diamond thermogravimetric/differential thermal analyser (Perkin-Elmer) to analyse the cabon content in carbon-coated TiP_2_O_7_ and NaTi_2_(PO_4_)_3_. X-ray photoelectron spectra (XPS) were collected by a Shimadzu/Kratos AXIS Ultra XPS spectrometer. All binding energies were referenced to the F 1*s* peak (from polyvinylidene fluoride) of 688.2 eV.

### Electrochemical measurements

Electrochemical measurements were carried on Solartron 1470E multi-channel potentiostats using either a two-electrode or a three-electrode cell set-up. For three-electrode set-up, an Ag/AgCl electrode (0.2 V versus SHE) and Pt gauze were employed as reference and counter electrodes, respectively. Electrode were prepared by casting slurries of active materials (75 wt%), Super P (15 wt%) and polyvinylidene fluoride (10 wt%) in *N*-methyl-2-pyrrolidinone on steel iron grid, and air drying at 80 °C for 12 h. Discs with diameter of 1.3 cm were cut for electrochemical tests. Mass loadings for the electrodes were determined by comparing the mass of the electrode with that of the original blank one.

### DFT calculations

The calculations were based on DFT method in the generalized gradient approximation with the Perdew, Burke and Ernzerhof functional^39^, as implemented in the Vienna *ab initio* Simulation Package, which employed a plane-wave basis^40^,^41^. The plane-wave energy cutoff was set to be 500.0 eV, and the electronic energy convergence was 10^−5^ eV. During relaxations, the force convergence for ions was 10^−2^ eV/Å. A Γ—centred 6 × 6 × 6 Monkhorst–Pack k-point mesh for primitive cell was used to sample the irreducible Brillouin zone. In InFe(CN)_6_, Fe ions locate in the octahedral environment and bond to carbon, and such carbon-coordinated Fe in a strong crystal field favours the low-spin configuration. To correctly characterize the localization of Fe *d*-electrons, GGA+U method with a Hubbard-type potential to describe the d-part of the Hamiltonian was applied. Previous studies using the U=3.0 eV for the low-spin Fe had demonstrated that GGA+U calculations could provide good predictions about the structural and electronic properties of HCF compounds^31^,^42^,^43^. Then, we set the U value of 3.0 eV for Fe atom during the calculations of structural relaxation and electronic structures.

### Data availability

The data that support the findings of this study are available from the corresponding author upon request.

## Additional information

**How to cite this article:** Chen, L. *et al.* Water-mediated cation intercalation of open-framework indium hexacyanoferrate with high voltage and fast kinetics. *Nat. Commun.* 7:11982 doi: 10.1038/ncomms11982 (2016).

## Supplementary Material

Supplementary Information Supplementary Figures 1-15, Supplementary Tables 1-5, Supplementary Notes 1-6, Supplementary Methods and Supplementary References.

## Figures and Tables

**Table 1:** Radii (unit: pm) of intercalants and the calculated binding energies (*E*_b_, unit: eV) when intercalant occupying different interstitial sites.

**Intercalant**	Radius*	***E***_**b**_				
		**8c**	**24d**	**32f(n)**	**32f(c)**	**48g**
Li^+^	76	−2.01	−**3.13**†	−3.05	−2.81	−**3.13**
Na^+^	102	−2.73	−**3.33**	−3.18	−3.17	−**3.33**
K^+^	138	−3.8	−3.41	−**3.86**	−3.85	−3.85
[Li-OH_2_]^+^	226			−4.00‡		
[Na-OH_2_]^+^	252			−3.94‡		
H_2_O	150			−0.67‡		

^*^The ionic radius of A^+^ is adapted from Shannon's report^17^. The radius of [A-OH_2_]^+^ is estimated by adding the radii of A^+^ and H_2_O*. r* (H_2_O) is supposed to be 150 pm.

^†^The bold font is used to emphasize the most stable site for each cation.

^‡^See details about how to calculate *E*_b_ for [A-OH_2_]^+^ and H_2_O in Supplementary Methods.
