# Supplementary material for: Water-mediated cation intercalation of open-framework indium hexacyanoferrate with high voltage and fast kinetics
Source: Nat Commun. 2016 Jun 20;7:11982. doi: 10.1038/ncomms11982 (PMC4915128; doi:10.1038/ncomms11982)
Supplement: Supplementary Information — Supplementary Figures 1-15, Supplementary Tables 1-5, Supplementary Notes 1-6, Supplementary Methods and Supplementary References. [file ncomms11982-s1.pdf]

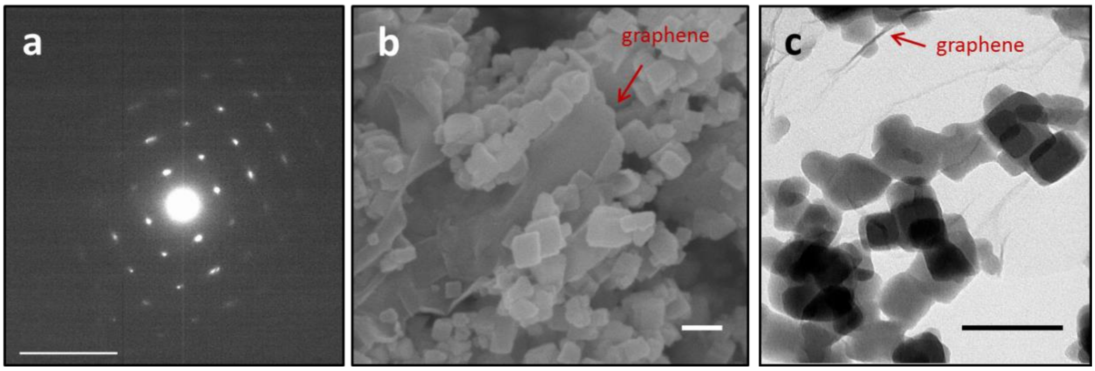

Supplementary Figure 1 | Structure of InHCF. a, Selected-area electron diffraction pattern of individual InHCF nanocube (scale bar 5 nm^{-1}). **b and c,** SEM and TEM image of InHCF/Gr (scale bar 100 nm).

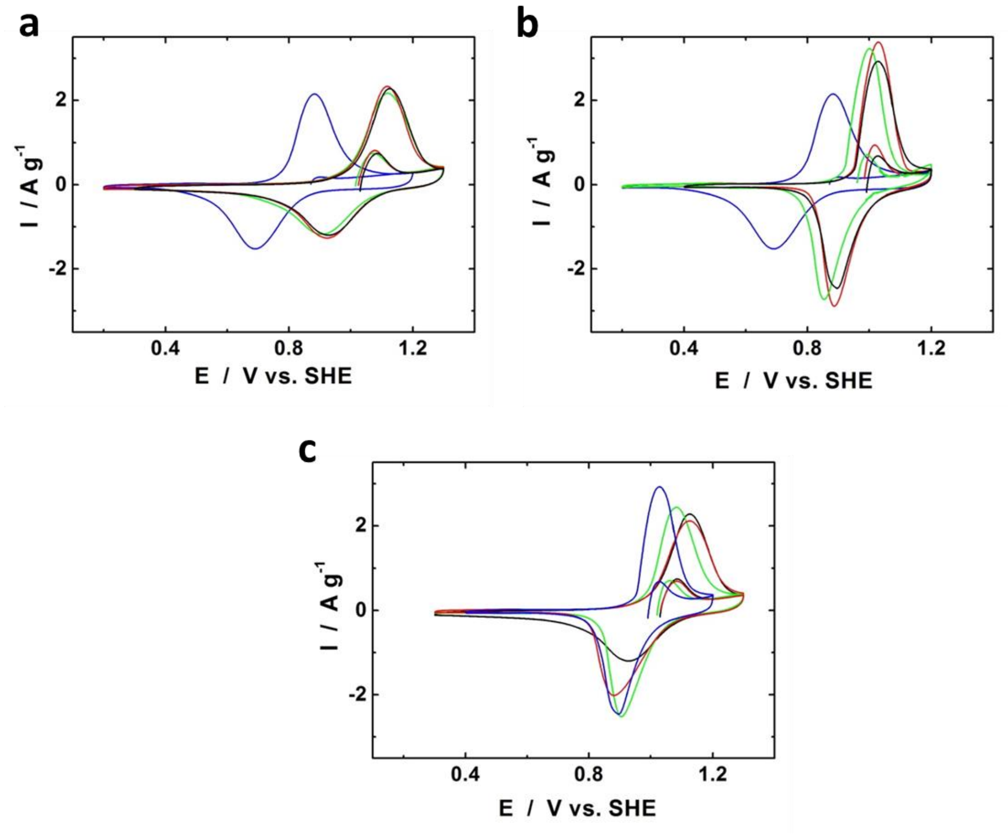

Supplementary Figure 2 | Cyclic voltammograms for InHCF/Gr in various electrolytes. Scan rate: 2 mV s^{-1}. All scans start from the open circuit voltage (E_{ocp}) of the electrodes in electrolytes. Blue line is corresponding to 0.5 M Li_2SO_4 in **a** and **b**; or 0.5 M K_2SO_4 in **c**. Green line is corresponding to 0.25 M Li_2SO_4 + 0.25 M Na_2SO_4 in **a**; or 0.25 M Li_2SO_4 + 0.25 M K_2SO_4 in **b**; or 0.25 M Na_2SO_4 + 0.25 M K_2SO_4 in **c**. Red line is corresponding to 0.1 M Li_2SO_4 + 0.4 M Na_2SO_4 in **a**; or 0.1 M Li_2SO_4 + 0.4 M K_2SO_4 in **b**; or 0.4 M Na_2SO_4 + 0.1 M K_2SO_4 in **c**. Black line is corresponding to 0.5 M Na_2SO_4 in **a** and **c**; or 0.5 M K_2SO_4 in **b**. The E_{ocp} of the electrodes in **a**, **b** and **c** follow the same order: black > red > green > blue. E_{ocp}, the open circuit potential of the as-prepared electrode in the electrolytes that is an important indicator of the chemical sates. From the E_{ocp}, we can see that InHCF/Gr is a compound with oxidation form.

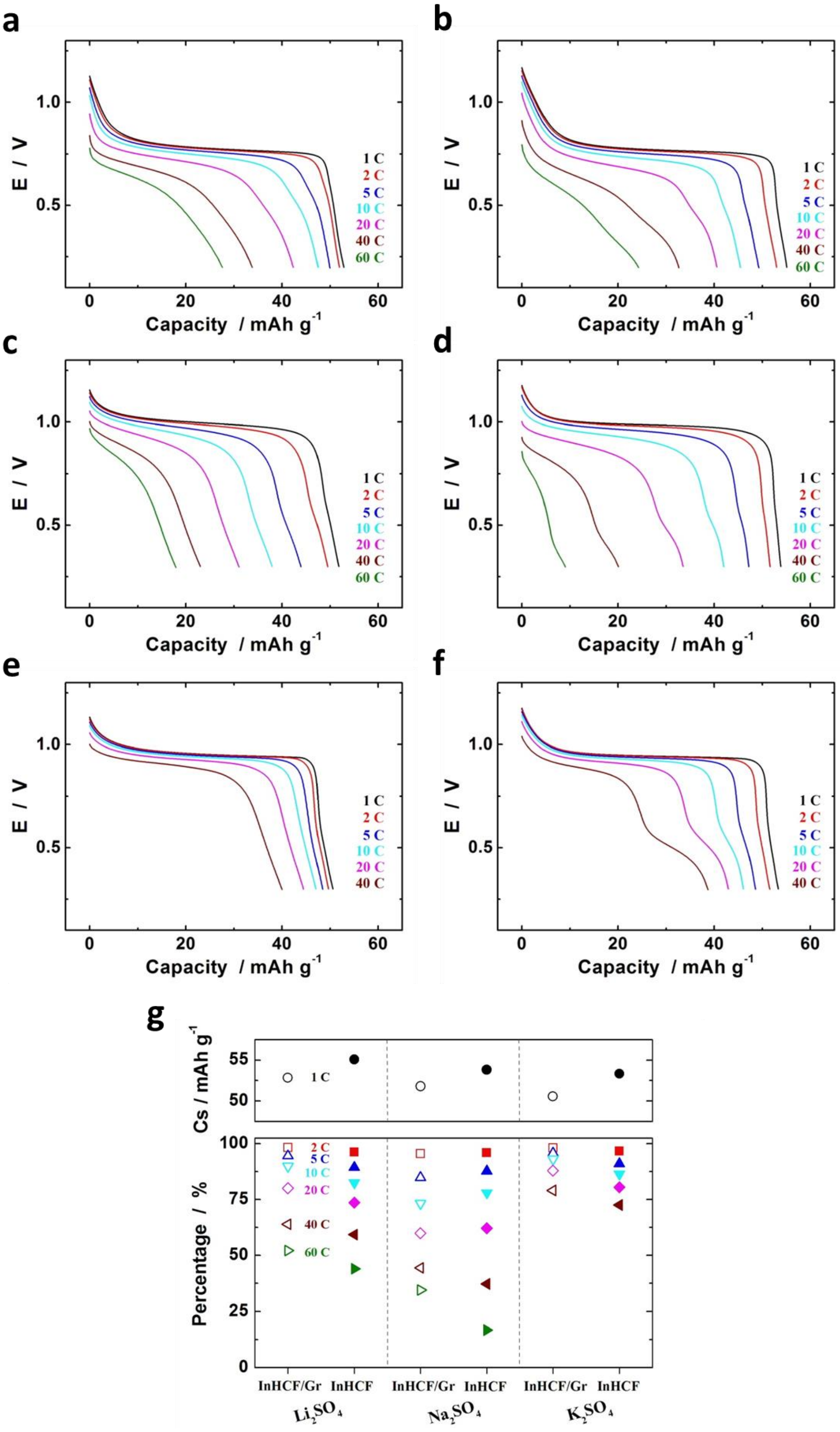
a
b
c
d
e
f
g
E / V
Capacity / mAh g-1
1 C
2 C
5 C
10 C
20 C
40 C
60 C
Cs / mAh g-1
Percentage / %
InHCF/Gr
InHCF
Li2SO4
Na2SO4
K2SO4

Supplementary Figure 3 | Comparison of rate capability between InHCF/Gr and InHCF. Rate capabilities of InHCF/Gr (**a**, **c**, **e**) and InHCF (**b**, **d**, **f**) in aqueous 0.5 M Li_2SO_4 (**a**, **b**), 0.5 M Na_2SO_4 (**c**, **d**) and 0.5 M K_2SO_4 (**e**, **f**). **g** is the summary of rate capabilities of InHCF/Gr and InHCF in different electrolytes (C_s is the specific discharging capacity, and percentage is the normalized discharging capacity which is compared with the discharging capacity measured at 1C rate). E (V) is normalized to SHE, and 1C equals to 60 mA g^{-1}.

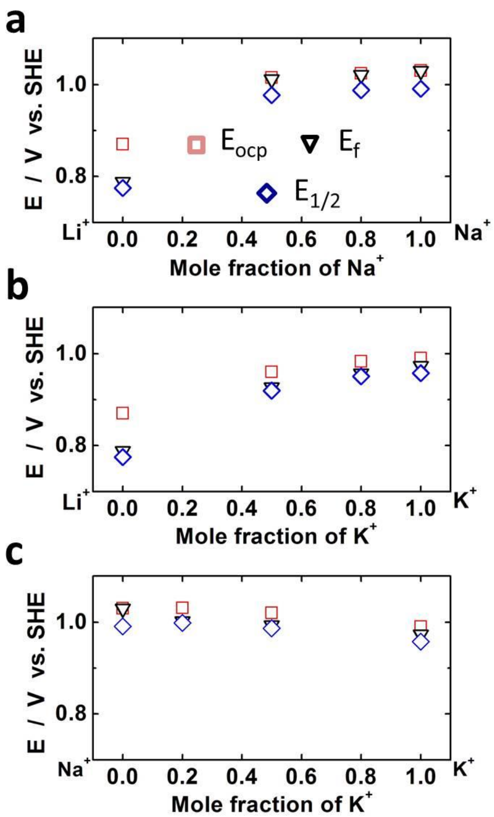

Supplementary Figure 4 | Plots of E_f, E_{ocp} and $E_{1/2}$ vs. the composition of mixed-ion electrolytes. **a**, Li^+/Na^+ mixed-ion electrolytes; **b**, Li^+/K^+ mixed-ion electrolytes; **c,** Na^+/K^+ mixed-ion electrolytes. E_f, formal potential, the average of the anode and cathode peak potentials (E_{pa} and E_{pc}); E_{ocp}, the open circuit potential of the as-prepared electrode in the electrolytes that is an important indicator of the chemical sates; $E_{1/2}$, the voltage that is measured when the electrode material has discharged 50% of its total capacity at a rate of 1C.

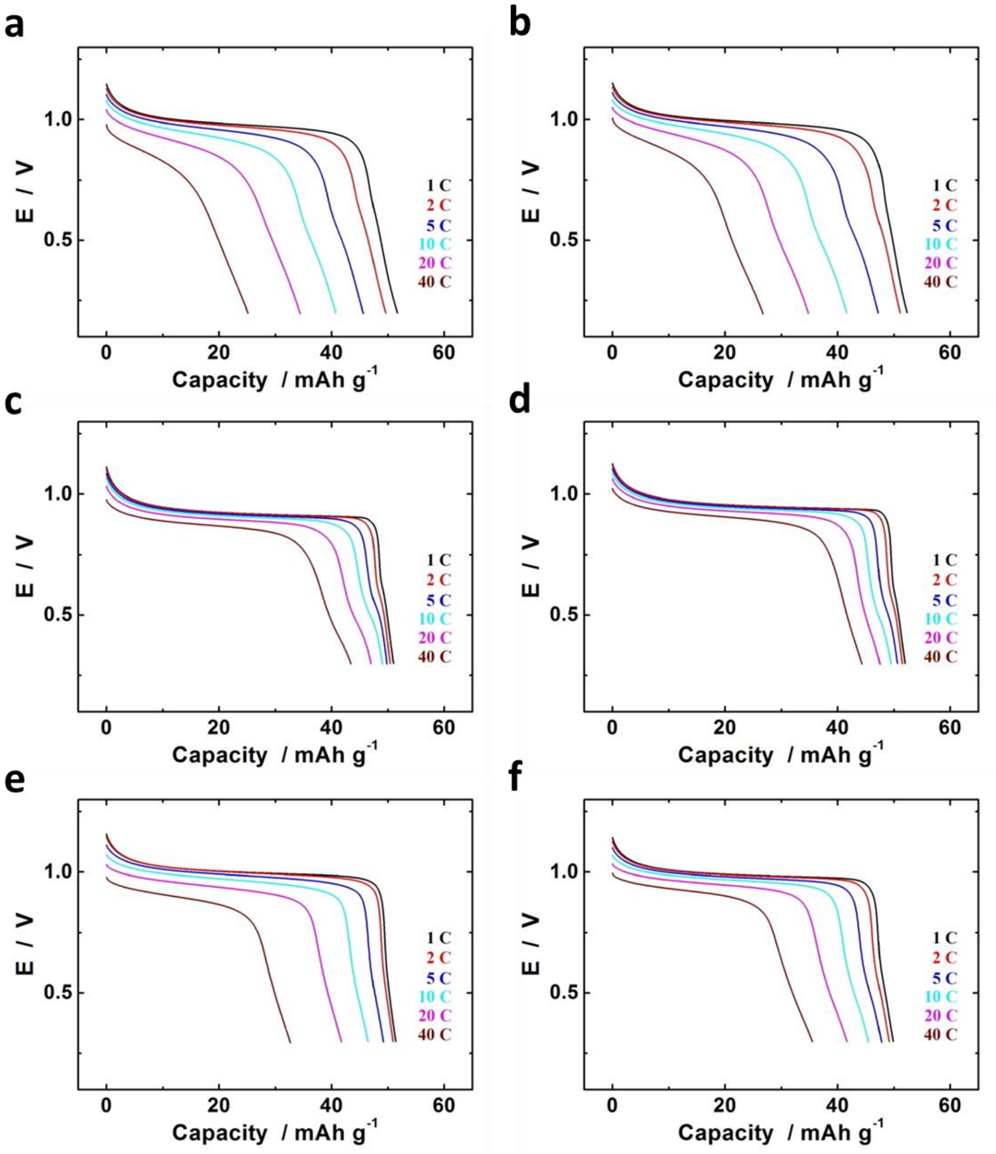

Supplementary Figure 5 | Rate capabilities of InHCF/Gr in various electrolytes. Rate capabilities of InHCF/Gr in aqueous 0.25 M Li_2SO_4+0.25 M Na_2SO_4 (**a**), 0.1 M Li_2SO_4+0.4 M Na_2SO_4 (**b**), 0.25 M Li_2SO_4+0.25 M K_2SO_4 (**c**), 0.1 M Li_2SO_4+0.4 M K_2SO_4 (**d**), 0.4 M Na_2SO_4+0.1 M K_2SO_4 (**e**), and 0.25 M Na_2SO_4+0.25 M K_2SO_4 (**f**). E (V) is normalized to SHE, and 1C equals to 60 mA g^{-1}.

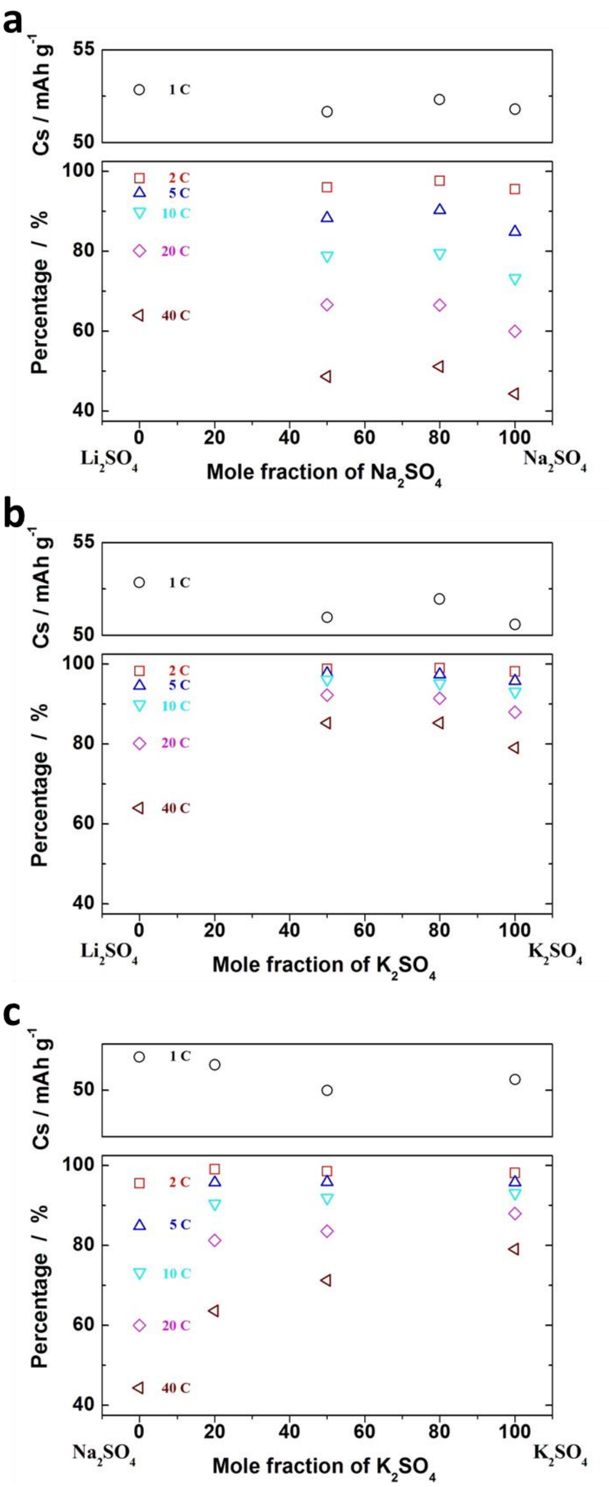

Supplementary Figure 6 | Summary of rate capabilities of InHCF/Gr in various mixed-ion electrolytes. **a**, Li^+/Na^+ mixed-ion electrolytes; **b**, Li^+/K^+ mixed-ion electrolytes; **c,** Na^+/K^+ mixed-ion electrolytes. C_s is the specific discharging capacity, and percentage is the normalized discharging capacity which is compared with the discharging capacity measured at 1C rate. 1C = 60 mA g^{-1}.

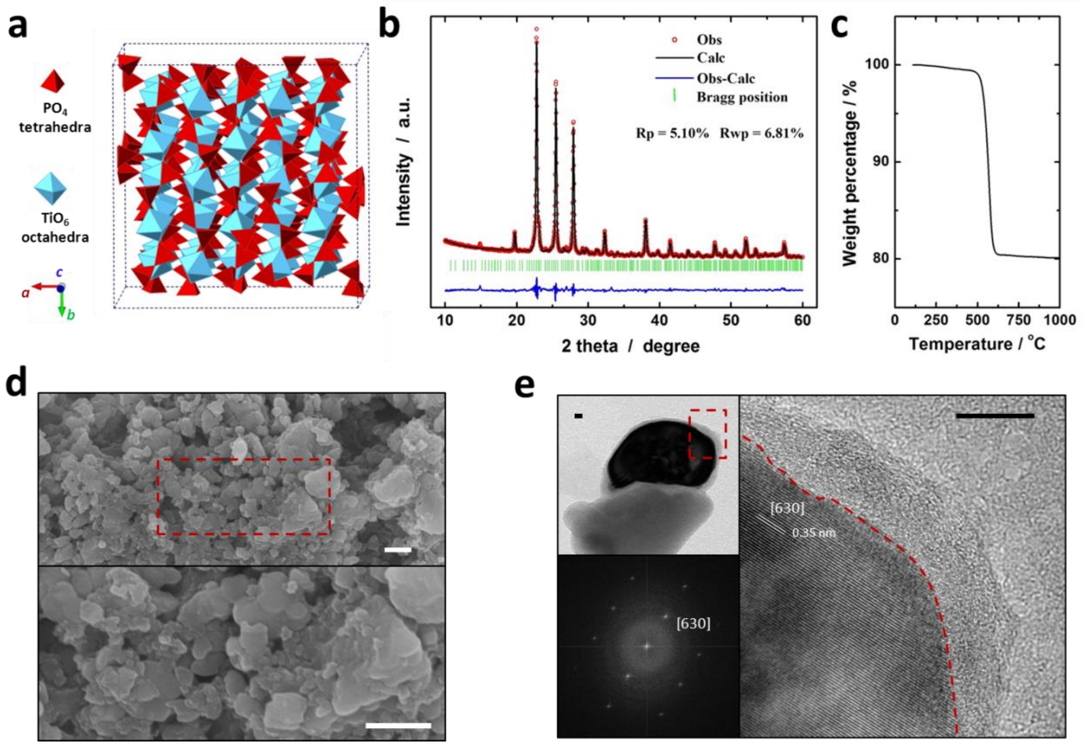

Supplementary Figure 7 | Cubic TiP_2O_7. **a**, crystal structure of cubic TiP_2O_7 with space group *Pa3*. **b**, Rietveld refinement of X-ray power diffraction pattern for carbon-coated TiP_2O_7. **c**, TGA curves of carbon-coated TiP_2O_7. **d**, SEM images of carbon-coated TiP_2O_7 with different magnification. Scale bar 400 nm. **e**, TEM image of TiP_2O_7 nanoparticles (upper left), high-resolution TEM image of selected section marked by red rectangle (right) and its corresponding FFT pattern (lower left). Scale bar 10 nm.

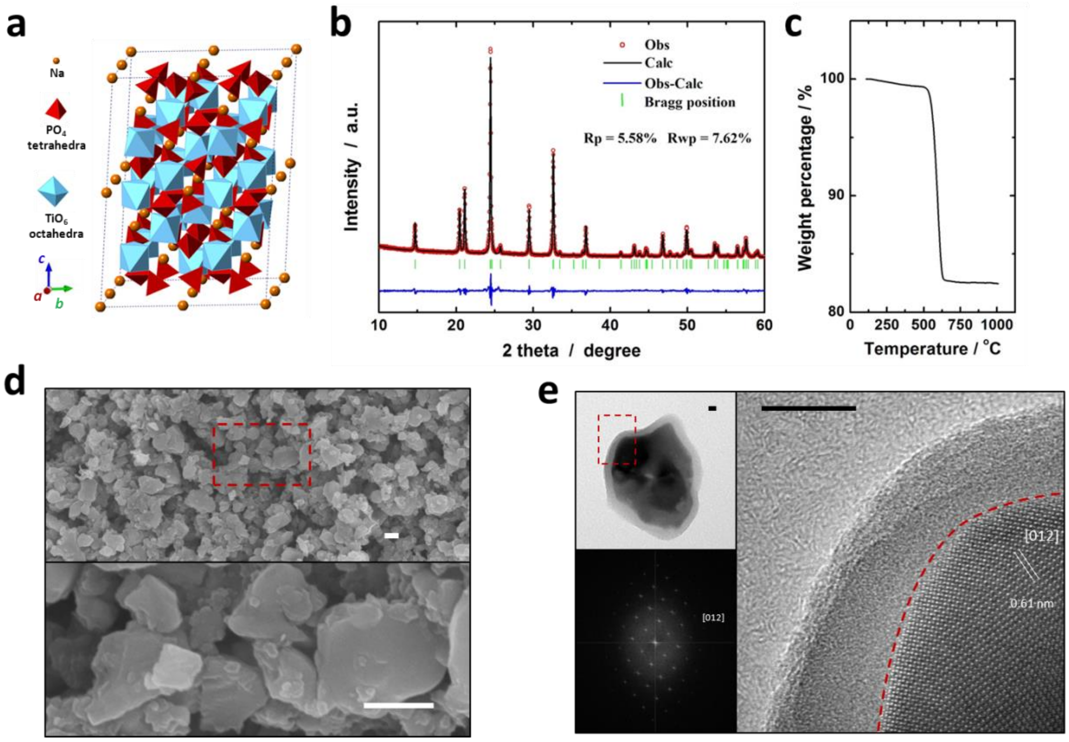

Supplementary Figure 8 | Rhombohedral $NaTi_2(PO_4)_3$. **a**, crystal structure of rhombohedral $NaTi_2(PO_4)_3$ with space group *R-3c*. **b**, Rietveld refinement of X-ray power diffraction pattern for carbon-coated $NaTi_2(PO_4)_3$. **c**, TGA curves of carbon-coated $NaTi_2(PO_4)_3$. **d**, SEM images of carbon-coated TiP_2O_7 with different magnification. Scale bar 400 nm. **e**, TEM image of $NaTi_2(PO_4)_3$ nanoparticles (upper left), high-resolution TEM image of selected section marked by red rectangle (right), and its corresponding FFT pattern (lower left). Scale bar 10 nm.

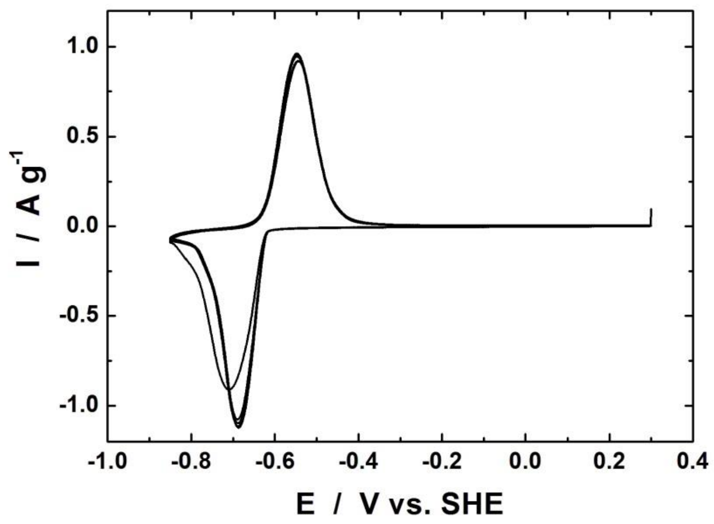

Supplementary Figure 9 | Cyclic voltammograms at a scan rate of 0.3 mV s^{-1} for carbon-coated $NaTi_2(PO_4)_3$ in 0.5 M Na_2SO_4 (initial 5 cycles). All electrode potentials are vs. standard hydrogen electrode (SHE).

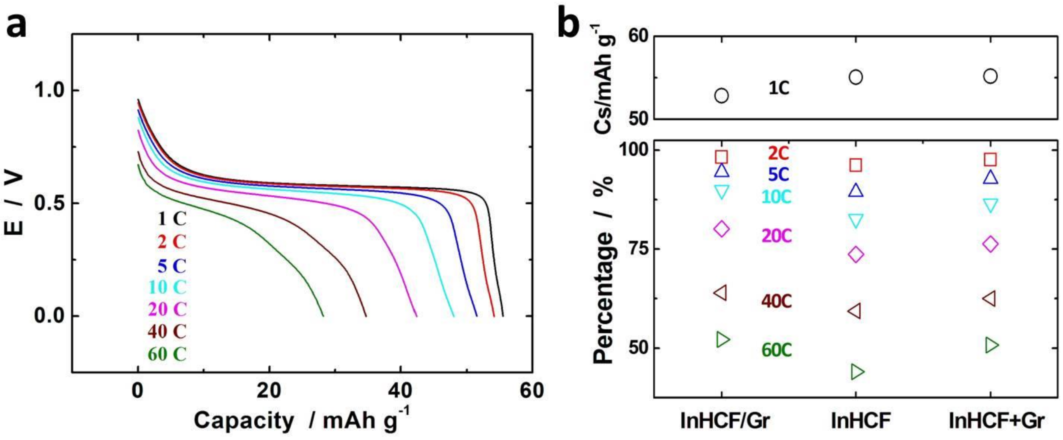

Supplementary Figure 10 | Rate capabilities of InHCF+Gr. a, discharging curves of InHCF+Gr in 0.5 M Li_2SO_4 at various rates. **b,** comparison of rate capabilities of InHCF/Gr, InHCF, InHCF+Gr in 0.5 M Li_2SO_4. (InHCF+Gr is the physical mixture of InHCF and graphene with the mass ratio of 10:1, C_s is the specific discharging capacity, and percentage is the normalized discharging capacity which is compared with the discharging capacity measured at 1C rate). E (V) is normalized to SHE, and 1C equals 60 mA g^{-1}.

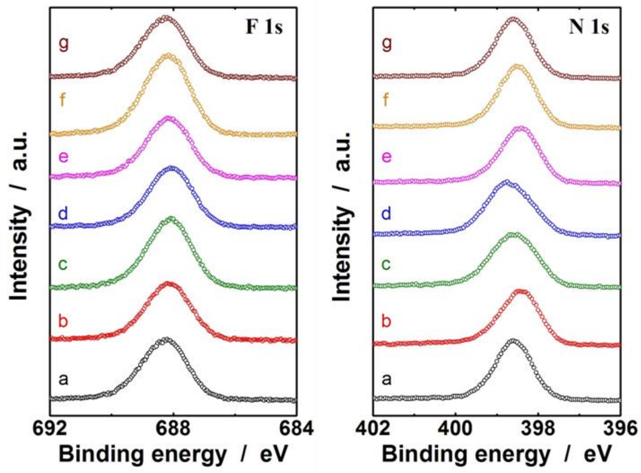

Supplementary Figure 11 | *Ex-situ* XPS spectra recorded from F 1s and N 1s core level of InHCF/Gr at different charging/discharging states. The explanations of different states are illustrated in the caption of Figure 5 in the manuscript.

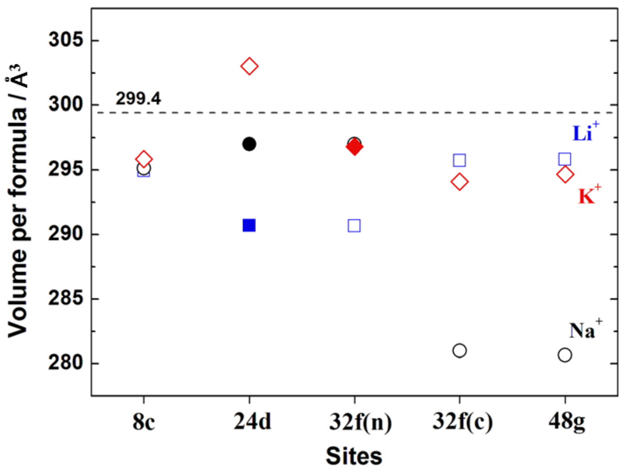

Supplementary Figure 12 | Volume of InHCF formula unit after A^+ (Li^+, Na^+ and K^+) occupying five different sites. The dashed line is the volume of InHCF formula unit with empty A^+ (299.4 $Å^3$). The most stable interstitial sites (24d sites for Li^+ and Na^+; 48g site for K^+) are marked by the solid symbols.

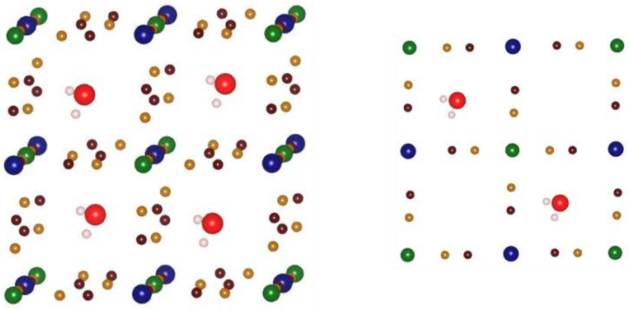

Supplementary Figure 13 | Crystal structures of InHCF with intercalation of H_2O. Green ball: Fe; blue ball: In; orange ball: C; wine ball: N; red ball: O; pink ball: H.

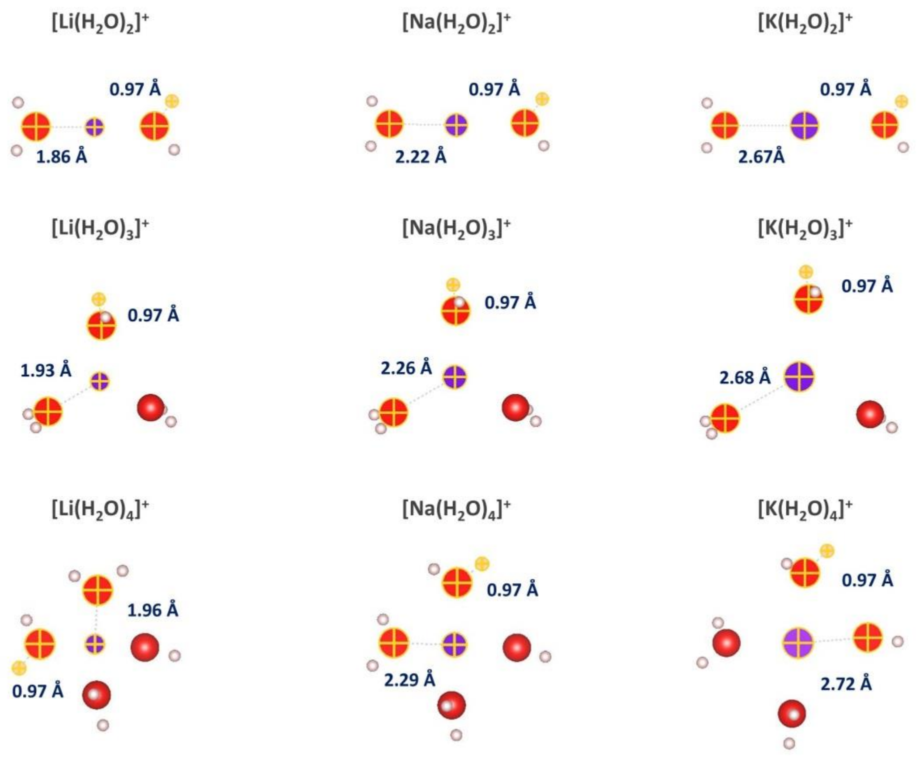

Supplementary Figure 14 | Geometries of solvated alkali cations (from 2-coordination to 4-coordination) determined by DFT calculations. $[A(H_2O)_2]^+$, $[A(H_3O)_2]^+$ and $[A(H_3O)_4]^+$ prefer linear, triangular and tetrahedral structure, respectively.

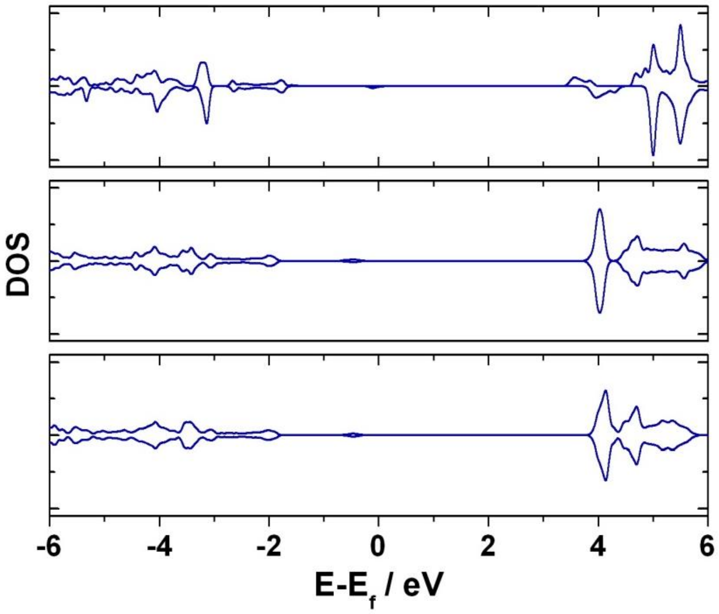

Supplementary Figure 15 | Electronic density of states projected on In atom in InHCF (top), NaInHCF (middle) and NaInHCF-H_2O (bottom).

Supplementary Table 1. Structural parameter of InHCF in the *Fm-3m* structure determined from Rietveld method using powder X-ray diffraction data (Rietveld) and *ab initio* calculations with the GGA+U approximation (GGA+U).

**Structural parameters**		**Value**	
		Rietveld	***GGA+U***
Lattice parameter	a = b = c	10.51 Å	10.62 Å
	α = β = γ	90°	90°
Bond distance	Fe-C	1.97 Å	1.91 Å
	In-N	2.13 Å	2.23 Å
	C-N	1.15 Å	1.17 Å
Angle	∠In-N-C	180°	180°
	∠Fe-C-N	180°	180°

Supplementary Table 2. Fractional coordinates of Fe, In, C and N of InHCF in the *Fm-3m* structure determined from Rietveld method using powder X-ray diffraction data (Rietveld) and *ab initio* calculations with the GGA+U approximation (GGA+U).

Atom	Wyckoff position	Method	x	y	z	Site occupancy
Fe	4a	Rietveld	0	0	0	1
		GGA+U	0	0	0	1
In	4b	Rietveld	0.5	0.5	0.5	1
		GGA+U	0.5	0.5	0.5	1
C	24e	Rietveld	0.1795	0	0	1
		GGA+U	0.1878	0	0	1
N	24e	Rietveld	0.2896	0	0	1
		GGA+U	0.2972	0	0	1

Supplementary Table 3. Energy densities & average operating voltagesof various RAMB. Energy density is calculated based on the total mass of active electrode materials.

RAMB type (cathode/anode)	Average operating voltage / V	Energy density / Wh kg^{-1}	Capacity retention / % (cycle no.)	Reference
**Aqueous Lithium-ion Batteries**				
$LiMn_2O_4/VO_2$	1.5	55	Failed (25)	[1]
$LiMn_2O_4/LiTi_2(PO_4)_3$	1.5	60	85 (50)	[2]
$LiMn_2O_4/TiP_2O_7$	1.4	59	37 (25)	[3]
$LiMn_2O_4/LiV_3O_8$	1.0	55	54 (100)	[4]
$LiFePO_4/LiTi_2(PO_4)_3$	0.9	50	90 (1000)	[5]
$LiCoO_2/LiV_3O_8$	1.0	35	36 (100)	[6]
$LiNi_{0.81}Co_{0.19}O_2/LiV_3O_8$	1.1	50	70 (30)	[7]
$LiMn_2O_4/Mo_6S_8$	1.7~1.8	84	78 (100)	[8]
$InHCF/TiP_2O_7$	1.2	41	n.a.	This work
**Aqueous Sodium-ion and Potassium-ion Batteries**				
$Na_{0.44}MnO_2/NaTi_2(PO_4)_3$	1.1	33	60 (700)	[9]
$Na_{0.44}MnO_2/AC$	0.9	< 40	≈ 90 (1000)	[10]
$Na_{0.44}[Mn_{0.66}Ti_{0.34}]O_2/NaTi_2(PO_4)_3$	< 1.2	not shown	89 (300)	[11]
$Na_{0.44}[Mn_{0.44}Ti_{0.56}]O_2/NaTi_2(PO_4)_3$	1.1	not shown	n.a.	[11] , [12]
$Na_2NiFe(CN)_6/NaTi_2(PO_4)_3$	1.27	43	88 (250)	[13]
$Na_2CuFe(CN)_6/NaTi_2(PO_4)_3$	1.4	48	90 (1000)	[14]
$Na_2CoFe(CN)_6/NaTi_2(PO_4)_3$	1 (1.7 and 0.4)	67	90 (800)	[15]
$K_{0.27}MnO_2/NaTi_2(PO_4)_3$	0.8	not shown	n.a.	[16]
$Na_2CuFe(CN)_6/NaMn_2(CN)_6$	0.95	27	> 90 (1000)	[17]
$KCuFe(CN)_6$/Ppy	0.9	5	> 90 (1000)	[18]
CoCuHCF/SNDI	< 1.1 V	26	86 (100)	[19]
$InHCF/NaTi_2(PO_4)_3$	1.55	54	n.a.	This work
**Aqueous Mixed-ion Batteries**				
$LiMn_2O_4/Na_{0.22}MnO_2$ (Li^+/Na^+)	0.6	21	83 (45)	Our group, [20]
$Na_{0.44}MnO_2/TiP_2O_7$ (Li^+/Na^+)	1.05	40	62 (50)	Our group, [20]
Ni_1Zn_1HCF/TiP_2O_7 (Li^+/K^+)	1.25	46	63 (50)	Our group, [21]
$Ni_1Zn_1HCF/NaTi_2(PO_4)_3$ (Na^+/K^+)	1.45	53	49 (50)	Our group, [21]
$InHCF/TiP_2O_7$ (Li^+/Na^+)	1.45	49	n.a.	This work
$InHCF/TiP_2O_7$ (Li^+/K^+)	1.40	48	n.a.	This work
$InHCF/NaTi_2(PO_4)_3$ (Na^+/K^+)	1.60	56	90 (200)	This work
$LiMn_2O_4$/Zn (Li^+/Zn^{2+})	1.7~1.8	50~80	95 (4000)	[22]
$LiFePO_4$/Zn (Li^+/Zn^{2+})	1.2	not shown	> 90 (100)	[22],[23]
$Na_{0.95}MnO_2$/Zn (Na^+/Zn^{2+})	1.5	78	92 (1000)	[24]
**Aqueous Zinc-ion Batteries**				
α-MnO_2/Zn	1.3	not shown	78 (100)	[25]
ZnHCF/Zn	1.7	100	81 (100)	Our group, [26]
CuHCF/Zn	1.7	not shown	96 (100)	[27]

Supplementary Table 4. Coulombic efficiency (CE) of InHCF/Na^{+}+K^{+}/$NaTi_2(PO_4)_3$ battery with cycles.

Cycle no.	CE/100%	Cycle no.	CE/100%	Cycle no.	CE/100%	Cycle no.	CE/100%
1	96.6	45	99.3	100	99.4	155	99.6
2	98.1	50	99.3	105	99.2	160	99.2
3	98.9	55	99.6	110	99.4	165	99.4
5	99.3	60	99.3	115	99.5	170	99.2
10	99.4	65	98.9	120	98.1	175	99
15	99.5	70	99.4	125	99.3	180	99.5
20	98.5	75	99.4	130	99.3	185	99.2
25	99.5	80	98.5	135	99.6	190	99.5
30	99.5	85	99.4	140	99.4	195	99.2
35	99.3	90	99.4	145	99.2	200	99.4
40	98.7	95	99.5	150	99.3		

Supplementary Table 5. Atomic concentration of different elements in InHCF/Gr during electrochemical cycling which are determined by XPS.

States	Atomic concentration / %			
	**Na**	**Fe**	**N**	**In**
a	7.84	10.15	68.86	13.15
b	5.39	11.40	70.62	12.59
c	4.29	10.73	71.14	13.84
d	3.12	12.09	71.23	13.56
e	6.37	10.64	69.50	13.49
f	7.22	11.45	67.32	14.01
g	7.72	10.00	69.44	12.84

Supplementary Note 1: the equal specific capacity of InHCF/Gr with Li^+, Na^+ and K^+.

Our InHCF/Gr is a cation-deficient (oxidation form) compound (see Supplementary Figure 2). An A^+-intercalation reaction that occurs at InHCF can be written as follows:

$$InHCF_{(Ox)} + nA^{+}_{(Aq)} + ne^{-} \leftrightarrow A_{n}InHCF_{(Red)}$$

It can be seen that $InHCF_{(Ox)}$ has the same molecular weight and $A_nInHCF_{(Red)}$ has different molecular weights with different A^+. Due to its same molecular weight, the specific discharging capacities of InHCF/Gr (oxidation form) with Li^+, Na^+ and K^+ are the same.

Supplementary Note 2: the coulombic efficiency and voltage charge limits of InHCF/Na^++K^+/$NaTi_2(PO_4)_3$ battery.

From the Supplementary Table 4, we can see that the coulombic efficiency is almost above 99 % at each cycle except some occasional cycles (e.g., 1th, 2th, 3th, 20 th, 65th, 80th, 120 th ….). So it is concluded that many side reactions (e.g., water electrolysis reactions) are eliminated during cycling despite the high voltage charge limit (1.83 V) for aqueous electrolytes.

Electrolysis of pure water into H_2 and O_2 at 298 K can be written as following Nernst equations:

O_2 evolution: $2H_2O\ (l) \rightarrow O_2\ (g) + 4H^+\ (aq) + 4e^-$ $E^{o}_{\mathrm{red}} = 1.23$ V vs. SHE

$$E(O_2) = E^{o}_{\mathrm{red}} - 4RT\ln[H^+]/4F = 1.23 - 0.059pH$$

H_2 evolution: $2H^+\ (aq) + 2e^- \rightarrow H_2\ (g)$ $E^{o}_{\mathrm{red}} = 0$ V vs. SHE

$$E(H_2) = E^{o}_{\mathrm{red}} - 2RT\ln[H^+]/2F = 0 - 0.059pH$$

So it can be seen that the reaction potentials of O_2 and H_2 evolution are pH-dependent. At pH = 7.0, the theoretical reaction potentials of O_2 and H_2 evolution are 0.82 V and –0.41 V vs. SHE, respectively. Our mixed-ion electrolytes as well as aqueous Li_2SO_4, Na_2SO_4 and K_2SO_4 are neutral electrolytes (pH = 7.0). For the mixed-ion batteries as shown in Figure 3 of the manuscript, the cut-off charging voltage for InHCF/Gr cathode is 1.2 V vs. SHE that is beyond 0.82 V. In terms of TiP_2O_7 and $NaTi_2(PO_4)_3$ anodes, their cut-off discharging voltages are –0.56 V and –0.65 V vs. SHE, respectively, which are lower than theoretical reaction potential of H_2 evolution (–0.41 V vs. SHE). Although the cut-off voltage limits for InHCF/Gr, TiP_2O_7 and $NaTi_2(PO_4)_3$ are beyond the theoretical reaction potentials of H_2/O_2 evolution, their coulombic efficiency are around 100% (see Figure 3 of the manuscript), suggesting that water electrolysis reactions (both H_2 and O_2 evolution) are negligible.

The electrolytes solutions before and after cycling have been analyzed by ICP-OES. It is found that after cycling, the total concentration of M^+ and N^+ of the electrolytes is 1.04 ± 0.01 M, almost the same as the one before cycling (1.01 ± 0.01 M).

Supplementary Note 3: the fading mechanism of InHCF/Na^++K^+/$NaTi_2(PO_4)_3$ battery.

Our previous studies and other groups have found that oxygen that could react with the discharged-stated anode electrode materials is the primary cause of the capacity fading upon cycling. Although our used electrolytes here are all deaerated solution, we found that TiP_2O_7 and $NaTi_2(PO_4)_3$ undergo very slight fading upon cycling in deaerated solution (see Supplementary Figure 9). It could be attributed to the trace of oxygen in the electrolytes. We also found that InHCF/Gr exhibits excellent cycle life (no obvious decaying is observed). So the fading of InHCF/Na^++K^+/$NaTi_2(PO_4)_3$ battery mainly comes from the $NaTi_2(PO_4)_3$ anode.

Supplementary Note 4: the role of graphene.

In our InHCF/Gr, the mass ratio between InHCF and graphene is about 10:1. We choose the physical mixture of InHCF and graphene (InHCF+Gr) to study the effect of graphene on the rate performance of InHCF. In InHCF+Gr, the mass ratio of InHCF and graphene is also fixed at 10:1. Supplementary Figure 10 shows that discharging curves of InHCF+Gr at various rates in 0.5 M Li_2SO_4. As shown, InHCF+Gr at various rates exhibits higher capacity retention than InHCF, indicating that graphene serves as a 3D electronic network to diminish the resistance between InHCF nanoparticles, which facilitates the electron transfer. And InHCF/Gr shows slightly higher capacity retention than InHCF+Gr. It can be explained by the fact that the average size of InHCF/Gr is smaller than that of InHCF (see the manuscript). The reason is that 2D graphene adsorbs InHCF nanocrystals during the nucleation stage of precipitation reaction, which blocks their further growth up. Similar electrochemical results are also found in Na_2SO_4, K_2SO_4, and mixed-ion electrolytes. So it is concluded that graphene not only acts as a moderator during nucleation, but also serves as an electronic network.

Supplementary Note 5: the role of mixed-ion electrolytes.

Our previous studies [20, 21] have shown that cubic TiP_2O_7 and rhombohedral $NaTi_2(PO_4)_3$ can be used as the anodes for AMIB, as a result of their specific ion-selectivity properties (TiP_2O_7, ion-selectivity toward Li^+ against Na^+ and K^+; $NaTi_2(PO_4)_3$, ion-selectivity toward Na^+ against K^+) and reasonable working voltages. When an intercalation reaction occurs at H with ion-selectivity towards A^+ as shown in equation (1), the reaction potential (*E*) can be calculated using the Nernst equation (2):

$$H_{(s)} + nA^{+}_{(aq)} + ne^{-} \leftrightarrow A_nH_{(s)} \quad (1)$$

$$E = E^{\ominus} + \frac{2.303\mathrm{RT}}{n\mathrm{F}}\lg(a^{n}_{\mathrm{A}^+} a_{\mathrm{H}}/a_{\mathrm{A}_n\mathrm{H}}) \quad (2)$$

where a_{A^+}, a_{H} and $a_{\mathrm{A}_n\mathrm{H}}$ refer to the activities of A^+, H and A^+-intercalated H (A_nH).

As the total concentration of M^+ and N^+ in our mixed-electrolytes is fixed at 1 M to ensure the equal conductivity of the electrolytes, the activity of a given cation in mixed-electrolytes (here we briefly consider it as the concentration of the cation) is below 1 M. Our previous electrochemical data have shown that *E* for TiP_2O_7 decrease with decreasing the Li^+/Na^+ or Li^+/K^+ ratio in mixed-electrolytes ($[Li^+] + [Na^+] = 1$ M and $[Li^+] + [K^+] = 1$ M), and *E* for $NaTi_2(PO_4)_3$ decrease with decreasing the Na/K^+ ratio in mixed-electrolytes ($[Na^+] + [K^+] = 1$ M) [20, 21]. Therefore, equations (1) and (2) are valid for the electrode materials with ion-selectivity property. In terms of electrode materials that allow for co-intercalation of two alkali cations such as InHCF, these equations are not valid.

The CV data in our previous paper have also show that Li^+ (not Na^+ and K^+) can be intercalated into TiP_2O_7, and Na^+ (not K^+) can be intercalated into $NaTi_2(PO_4)_3$ [20, 21]. The ion-selective properties of above TiP_2O_7 and $NaTi_2(PO_4)_3$ can be easily explained by the steric effect: Na^+ and K^+ are too large to enter into TiP_2O_7 three-dimensional framework while K^+ is also too large for $NaTi_2(PO_4)_3$. For AMIB, one important characteristic is that the total concentration of M^+ and N^+ in the electrolytes is fixed during charging/discharging, but the M^+/N^+ ratio is changed. We have demonstrated this point in the paper [20].

From our previous [20, 21] and current studies, the A^+-intercalation voltages for TiP_2O_7 and $NaTi_2(PO_4)_3$ (anode materials) are decreased by 40 ~ 50 mV when using mixed cations as electrolytes instead of single cation. Here, the working voltages of InHCF/Gr (cathode material) are increased by 200 mV (Li^+/Na^+) and 150 mV (Li^+/K^+) in the mixed electrolytes when compared to electrolytes containing only Li^+. So compared to InHCF/Li^+/TiP_2O_7 battery, the increases of voltage output for InHCF/Li^++Na^+/TiP_2O_7 (250 mV) and InHCF/Li^++K^+/TiP_2O_7 (200 mV) batteries are mainly from the InHCF cathode. The working voltage of InHCF/Gr in Na^+/K^+-mixed electrolytes is almost identical to the one in Na_2SO_4. When compared to InHCF/Na^+/$NaTi_2(PO_4)_3$ battery, the slight increase of voltage output (ca. 50 mV) for InHCF/Na^++K^+/$NaTi_2(PO_4)_3$ battery comes from the $NaTi_2(PO_4)_3$ anode.

Supplementary Note 6: water-mediated cation intercalation of InHCF.

Depending on the number of co-ordinated water, $[A(H_2O)_n]^+$ solvated ions has different geometries. Supplementary Figure 14 displays the geometries of solvated ions (from 2-coordination to 4-coordination) determined by DFT calculations. As shown, $[A(H_2O)_2]^+$, $[A(H_3O)_3]^+$ and $[A(H_3O)_4]^+$ prefer linear, triangular and tetrahedral structure, respectively. The bond distances of Li-O, Na-O and K-O are 1.9 Å, 2.2 Å and 2.7 Å, respectively, which are very close to the experimental values (2.0 Å, 2.3 Å and 2.7 Å) in reference [Marcus, Y. *Chem. Rev.* **88,** 1475–1498 (1988).]. When $[Li\text{-}OH_2]^+$ or $[Na\text{-}OH_2]^+$ is intercalated into InHCF, the bond distance of Li-O or Na-O in InHCF is 1.88 Å (or 2.24 Å), similar to the one in $[A(H_2O)_n]^+$ solvated ions.

Meanwhile, the sizes of all solvated ions (from 2-coordination to 4-coordination) shown in the Supplementary Figure 14 are estimated to be above 5.2 Å. In the cubic structure of InHCF as shown in Fig. 6a of the manuscript, A^+ can occupy five possible interstitial sites. Among them, 8c site provides the largest free space. To avoid significant expansion of the lattice framework, the radius of guest ion to occupy the 8c site should be limited to 260 pm. So it is concluded that above solvated ions as well as $[K\text{-}OH_2]^+$ (radius 288 pm) can not be intercalated into InHCF.

Supplementary Methods

DFT calculation.

During the charging and discharging of an alkali-ion battery, an alkali A^+ is inserted or extracted from a host crystal structure A_nH. For a battery that operates by shuttling A^+ ions between the cathode and a pure alkali metal anode, the overall cell reaction can be written as follows:

$$H_{(s)} + nA_{(s)} \leftrightarrow A_nH_{(s)} \quad (1)$$

The forward reaction is the cell discharging reaction, while the reverse is the cell charging reaction. The average intercalation potential V vs. A/A^+ can then be calculated using the following equation [28, 29]:

$$V = -\frac{E_b}{ne} = -\frac{E(\mathrm{A}_n\mathrm{H}) - E(\mathrm{H}) - nE(\mathrm{A})}{ne} \quad (2)$$

Where E_b is the binding energy between A^+ ion and H, $E(A_nH)$, E(H) and E(A) are the total energies of A_nH, H and metallic A which are calculated using DFT, and e is the absolute value of the electron charge. In aqueous alkali-ion battery, m/n H_2O accompany the insertion and extraction of one A^+ during entire charge and discharge process. So the overall cell reaction can be written by equation (3):

$$H_{(s)} + nA_{(s)} + mH_2O \leftrightarrow A_n(H_2O)_mH_{(s)} \quad (3)$$

The average intercalation potential V vs. A/A^+ can then be calculated using the following equation:

$$V = -\frac{E_b}{ne} = \frac{E(\mathrm{A}_n(\mathrm{H_2O})_m\mathrm{H}) - E(\mathrm{H}) - nE(\mathrm{A}) - mE(\mathrm{H_2O})}{ne} \quad (4)$$

Where E_b is the binding energy between $[A\text{-}(OH_2)_{m/n}]^+$ ion and H, $E(A_n(H_2O)_mH)$ and $E(H_2O)$ are the total energies of $A_n(H_2O)_mH$ and water which are calculated using DFT. In this work, we employ m = n = 1 to calculate the average intercalation potential and E_b for $[Li\text{-}OH_2]^+$ and $[Na\text{-}OH_2]^+$, and m = 1, n = 0 to calculate E_b for H_2O. The calculated energies of InHCF, metallic Li, Na, K and H_2O are as follows: E(InHCF) = –108.71 eV; E(Li) = –1.89 eV; E(Na) = –1.30 eV; E(K) = –1.04 eV; $E(H_2O)$ = –14.22 eV.

Supplementary References

1. Li, W., Dahn, J. R. & Wainwright, D. S. Rechargeable lithium batteries with aqueous electrolytes. *Science* **264,** 1115–1118 (1994).
2. Luo, J. Y. & Xia, Y. Y. Aqueous lithium-ion battery $LiTi_2(PO_4)_3/LiMn_2O_4$ with high power and energy densities as well as superior cycling stability. *Adv. Funct. Mater.* **17,** 3877–3884 (2007).
3. Wang, H. B., Huang, K. L., Zeng, Y. Q., Yang, S. & Chen, L. Q. Electrochemical properties of TiP_2O_7 and $LiTi_2(PO_4)_3$ as anode material for lithium ion battery with aqueous solution electrolyte. *Electrochim. Acta.* **52,** 3280–3285 (2007).
4. Wang, G. J., Zhang, H. P., Fu, L. J., Wang, B. & Wu, Y. P. Aqueous rechargeable lithium battery (ARLB) based on LiV_3O_8 and $LiMn_2O_4$ with good cycling performance. *Electrochem. Commun.* **9,** 1873–1876 (2007).
5. Luo, J. Y., Cui, W. J., He, P. & Xia, Y. Y. Raising the cycling stability of aqueous lithium-ion batteries by eliminating oxygen in the electrolyte. *Nat. Chem.* **2,** 760–765 (2010).
6. Wang, G. J. *et al*. An aqueous rechargeable lithium battery with good cycling performance. *Angew. Chem. Int. Ed.* **46,** 295–297 (2007).
7. Kohler, J., Makihara, H., Uegaito, H., Inoue, H. & Toki, M. LiV_3O_8: characterization as anode material for an aqueous rechargeable Li-ion battery system. *Electrochim. Acta.* **46,** 59–66 (2000).
8. Suo, L. M. *et al*. “Water-in-salt” electrolyte enables high-voltage aqueous lithium-ion chemistries. *Science* **350,** 938–943 (2015).
9. Li, Z., Young, D., Xiang, K., Caeter, W. C. & Chiang, Y. M. Towards high power high energy aqueous sodium-Ion batteries: The $NaTi_2(PO_4)_3/Na_{0.44}MnO_2$ system. *Adv. Energy. Mater.* **3,** 290–294 (2013).
10. Whitacre, J. F., Tevar, A. & Sharma, S. $Na_4Mn_9O_{18}$ as a positive electrode material for an aqueous electrolyte sodium-ion energy storage device. *Electrochem. Commun.* **12,** 463–466 (2010).

1\. Wang, Y. S. *et al*. A novel high capacity positive electrode material with tunnel-type structure for aqueous sodium-ion batteries. *Adv. Energy Mater.* **5,** 1501005 (2015).

11\. Wang, Y. S. *et al*. Ti-substituted tunnel-type $Na_{0.44}MnO_2$ oxide as a negative electrode for aqueous sodium-ion batteries. *Nat. Commun.* **6,** 6401 (2015).

12\. Wu, X. Y., Cao, Y. L., Ai, X. P., Qian, J. F. & Yang, H. X. A low-cost and environmentally benign aqueous rechargeable sodium-ion battery based on $NaTi_2(PO_4)_3$–$Na_2NiFe(CN)_6$ intercalation chemistry. *Electrochem. Commun.* **31,** 145–148 (2013).

13\. Wu, X. Y. *et al*. Energetic aqueous rechargeable sodium-ion battery based on $Na_2CuFe(CN)_6$-$NaTi_2(PO_4)_3$ intercalation chemistry. *ChemSusChem* **7,** 407–411 (2014).

14\. Wu, X. Y. *et al*. Vacancy-free Prussian blue nanocrystals with high capacity and superior cyclability for aqueous sodium-ion batteries. *ChemNanoMat* **1,** 188–193 (2015).

15\. Liu, Y. *et al*. High-performance aqueous sodium-ion batteries with $K_{0.27}MnO_2$ cathode and their sodium storage mechanism. *Nano Energy* **5,** 97–104 (2014).

16\. Pasta, M. *et al*. Full open-framework batteries for stationary energy storage. *Nat. Commun.* **5,** 3007 (2014).

17\. Pasta, M., Wessells, C. D., Huggins, R. A. & Cui. Y. A high-rate and long cycle life aqueous electrolyte battery for grid-scale energy storage. *Nat. Commun.* **3,** 1149 (2012).

18\. Kim, D. J. *et al*. An aqueous sodium ion hybrid battery incorporating an organic compound and a Prussian blue derivative. *Adv. Energy Mater.* **4,** 1400133 (2014).

19\. Chen, L. *et al*. New-concept batteries based on aqueous Li^+/Na^+ mixed-ion electrolytes. *Sci. Rep.* **3,** 1946 (2013).

20\. Chen, L., Zhang, L. Y., Zhou, X. F. & Liu, Z. P. Aqueous batteries based on mixed monovalence metal Ions: a new battery family. *ChemSusChem*, **7,** 2295–2302 (2014).

21\. Yan, J. *et al*. Rechargeable hybrid aqueous batteries. *J. Power Sources* **216,** 222–226 (2013).

22\. Zhang**,** H. P., Wu**,** X., Yang**,** T., Liang, S. S. & Yang, X. J. Cooperation behavior between heterogeneous cations in hybrid batteries. *Chem. Commun.* **49,** 9977–9979 (2013).

23\. Zhang, B. H. *et al*. An aqueous rechargeable battery based on zinc anode and $Na_{0.95}MnO_2$. *Chem. Commun.* **50,** 1209–1211 (2014).

24\. Xu, C. J., Li, B. H., Du, H. D. & Kang, F. Y. Energetic zinc ion chemistry: the rechargeable zinc ion battery. *Angew. Chem. Int. Ed.* **51,** 933–935 (2012).

25\. Zhang, L. Y., Chen, L., Zhou, X. F. & Liu, Z. P. Towards high-voltage aqueous metal-ion batteries beyond 1.5 V: The zinc/zinc hexacyanoferrate system. *Adv. Energy Mater.* **5,** 1400930 (2015).

26\. Trocoli, R. & Mantia., F. L. An aqueous zinc-ion battery based on copper hexacyanoferrate. *ChemSusChem* **8,** 481–485 (2015).

27\. Aydinol, M. K., Kohan, A. F., Ceder, G., Cho, K. & Joannopoulos, J. *Ab initio* study of lithium intercalation in metal oxides and metal dichalcogenides. *Phys. Rev. B* **56,** 1354–1365 (1997).

28\. Ong, S. P. *et al*. Voltage, stability and diffusion barrier differences between sodium-ion and lithium-ion intercalation materials. *Energy Environ. Sci.* **4,** 3680–3688 (2011).
